# Skin fairness is a better predictor for impaired physical and mental health than hair redness

**DOI:** 10.1038/s41598-019-54662-5

**Published:** 2019-12-02

**Authors:** Jaroslav Flegr, Kateřina Sýkorová

**Affiliations:** 1 Division of Biology, Faculty of Science, Charles University, Prague, Viničná 7 128 44 Czech Republic; 2 Applied Neurosciences and Brain Imagination, National Institute of Mental Health, Klecany, 250 67 Czech Republic

**Keywords:** Epidemiology, Risk factors, Anxiety

## Abstract

About 1–2% of people of European origin have red hair. Especially female redheads are known to suffer higher pain sensitivity and higher incidence of some disorders, including skin cancer, Parkinson’s disease and endometriosis. Recently, an explorative study performed on 7,000 subjects showed that both male and female redheads score worse on many health-related variables and express a higher incidence of cancer. Here, we ran the preregistered study on a population of 4,117 subjects who took part in an anonymous electronic survey. We confirmed that the intensity of hair redness negatively correlated with physical health, mental health, fecundity and sexual desire, and positively with the number of kinds of drugs prescribed by a doctor currently taken, and with reported symptoms of impaired mental health. It also positively correlated with certain neuropsychiatric disorders, most strongly with learning disabilities disorder and phobic disorder in men and general anxiety disorder in women. However, most of these associations disappeared when the darkness of skin was included in the models, suggesting that skin fairness, not hair redness, is responsible for the associations. We discussed two possible explanations for the observed pattern, the first based on vitamin D deficiency due to the avoidance of sunbathing by subjects with sensitive skin, including some redheads, and second based on folic acid depletion in fair skinned subjects, again including some (a different subpopulation of) redheads. It must be emphasized, however, that both of these explanations are only hypothetical as no data on the concentration of vitamin D or folic acid are available for our subjects. Our results, as well as the conclusions of current reviews, suggest that the new empirical studies on the concentration of vitamin D and folic acids in relation to skin and hair pigmentation are urgently needed.

## Introduction

About 1–2% of subjects of European origin, but for example 6–13% of people in Wales, Scotland and Ireland, have red hair, depending on the definition of red hair, method of measurement and age-cohort^1,2^. It has been suggested that this trait spread in Europe by frequency-dependent selection acting in favor of women with rare phenotypes^3^. It has been proposed recently that the red hair phenotype, in contrast to the blond hair phenotype, is kept steadily rare by some form of natural selection acting in the direction opposite to sexual selection^4^. People with red hair, pale skin color and the inability to tan have the highest risk of developing melanoma^5^. The red hair/fair skin phenotype is usually, but not always, the result of inactivation of the gene for the melanocortin 1 receptor (MC1R), the product of which, a cyclic ATP-stimulating G protein, regulates the synthesis of pigments. Individuals with low receptor activity produce the red/yellow pheomelanin pigment instead of the brown/black eumelanin pigment^6^. Pheomelanin has minimal protective activity against ultraviolet radiation. Moreover, pheomelanin, or products of its metabolism, increases by some unknown UV-radiation-independent mechanism oxidative DNA damaging^7–10^. This again strongly increases the risk of melanoma in both sun-exposed and non-exposed skin^10^. Several studies also showed that redheads have an increased risk of Parkinson’s disease^11^, especially of the early-onset form of this disorder^12^. This probably explains the observed association between Parkinson’s disease and incidence of melanoma cancer. A genetic association study^13^ as well as experiments performed with animal models of the redhead phenotype^14^ showed that the MC1R plays a crucial role in the association between red hair and Parkinson’s disease. Anecdotal observations as well as several case-controls studies suggested a higher pain sensitivity^15^, an increased risk of endometriosis^16,17^, and an increased bleeding tendency^18,19^ of red haired women. It must be mentioned, however, that the observed effect sizes are usually small and that some studies provided negative results.

Recently, a large anonymous cross-sectional study performed on a cohort of nearly 7,000 internet users showed that the intensity of redness of hair correlated, mostly negatively, with several health-related variables^4^. For example, women with more intense redness of hair reported significantly worse physical health, mental health, more frequent heart and vascular system problems, metabolic problems, cancer problems, fertility problems, genitourinary problems, psychiatric problems, musculoskeletal problems, and sexual function problems. Men with more intensive redness of hair reported worse mental health, a higher number of different drugs prescribed by medical doctors taken per day, and a higher number of “different herbs, food supplements, multivitamins, superfoods etc.” they were taking per day. Red headed women reported more frequent diagnoses of cervical uterine cancer, cervical uterine precancerosis, ovarian cancer and other cancer. Both male and female redheads reported more frequent diagnoses of colorectal cancer.

Redheads, especially men, are subjects of many prejudices^20^ and also often victims of bullying^21^. Typically, redheads receive negative treatment as children and consequently they experience lowered self-esteem^21^. It was shown that subjects who reported having been bullied in childhood also reported significantly more mental health problems in adulthood, which, secondarily, could be reflected by the occurrence of various psychosomatic symptoms^22–24^. The main purpose of the present preregistered cross-sectional study was to search for indices of impaired mental health of subjects with a higher intensity of hair redness. Therefore, we asked the anonymous participants of the present study to rate the intensity of depression, anxieties, manias, phobias, obsessions, auditory hallucination, visual hallucinations, burnout syndrome, and headache. We also asked them to report the occurrence of any mental health disorder diagnosed by a medical doctor by checking it in a list of 26 neuropsychiatric disorders. In the confirmatory part of the study, we searched for an association between intensity of hair redness and mental and physical health, while in the exploratory part of the study we searched for possible reasons for observed associations. Namely, we tested whether the association became stronger, or rather disappeared, when the effects of potentially confounding variables, such as darkness of hair, eye, and skin, was controlled.

## Material and Methods

This is a preregistered case-control study Flegr, J. (2017, May 1) “Red hair color phenotype has negative effects on physical and mental health”, osf.io/f67m8. The experimental setup including the stopping rule, three hypotheses to be tested and statistical tests for testing these particular hypotheses were registered before the start of data collecting.

### Subjects

The internet questionnaire was distributed as a Qualtrics survey. The subjects were invited to participate in the study using a Facebook-based snowball method^25^. In short, potential volunteers, mostly the members of the “Lab bunnies” community, an 18,000-member group of Czech and Slovak nationals willing to take part in evolutionary psychology experiments, and their Facebook friends, were invited (using 5–10 different posts on the Lab bunnies timeline) to participate in anonymous studies “investigating for example philosophical problems of concept of self, testing evolutionary hypothesis concerning beauty of flowers, and studying moral dilemmas concerning autonomous cars”. Neither the color and darkness of hair or darkness of eyes or skin, nor the health-related problems, were explicitly mentioned in the informed consent. The questionnaire was written in Czech and due to the highly ethnically homogeneous population of the Czech Republic, nearly 100% of participants were Caucasian. The respondents were not paid for their participation in the study; however, after finishing the 60-minute questionnaire, they were provided information about the results of related studies and their own results from two personality tests that were part of the questionnaire (CSIV, Big Five). At the first screen of the test, the participants were provided with the following information and were asked to provide their informed consent to participate in the study by pressing special button: “The study is anonymous and obtained data will be used exclusively for scientific purposes. Your cooperation in the project is voluntary and you can terminate it at any time by closing this web page. You can also skip any uncomfortable questions; however, complete data is most valuable. If you agree to participate in the research press the “Next” button”. Only the subjects who provided their informed consent were allowed to participate in the study. Some pages of the questionnaire contained the Facebook share button. These buttons were pressed by more than 170 participants, which resulted in obtaining data from 4,624 responders in total between 17^th^ May 2017 and 31^st^ December 2017. All methods were performed in accordance with the relevant guidelines and regulations. The project, including the method of obtaining electronic informed consent to participate in this anonymous study from all participants, was approved by the IRB of the Faculty of Science, Charles University (Komise pro práci s lidmi a lidským materiálem Přírodovědecké Fakulty Univerzity Karlovy) - No. 2017/25.

### Questionnaire

The electronic questionnaire consisted of several parts that concerned various unrelated projects on evolutionary psychology, philosophy and ethics. In the present study, we inspected and analyzed only the responses to the questions concerning health and pigmentation of hair, eye, and skin. Namely, we analyzed the following outcome variables: binary variables: incidence of 26 mental health disorders diagnosed by medical doctors, including the category “other disorder” (only 22 disorders reported by more than 9 subjects were included into the analyses; ordinal (0–5 scale): How they have felt (physically) for the last two years, How they have felt (mentally) for the last two years, How many kinds of drugs prescribed by a medical doctor for mental health problems were they taking the last month, How many kinds of drugs prescribed by a doctor for other health problems were they taking the last month, How many children do they have; semi-continuous (1–100 scales) variables: How much do they suffer with anxieties, phobias, depressions, manias, obsessions, auditory hallucinations, visual hallucinations, burnout syndrome, and headache. Using the same 1–100 scale they also answered the questions - How intensively they are sexually attracted to opposite sex people, How intensively they are sexually attracted to same-sex people, (the higher of these two responses was considered as the intensiveness of sexual desire). Predictors, namely the intensity of natural hair redness, natural hair darkness, eye darkness, and natural skin darkness – were rated by responders using the six-step rating scales 0–5. The scale for redness of hair was anchored with absolutely non-red (code 0)–brightly red (code 5), and other scales (darkness of hair, eye, and skin) were anchored with very light (code 0)–very dark (code 5). We also monitored potential confounding variables including sex, age, and size of place of living (six categories: <1000 inhabitants,1000–5000, 5000–50,000, 50,000–100,000, 100,000–500,000, Prague or Bratislava).

### Statistical analysis

Before any analyses, records from about 2% of subjects who did not check the correct answers to two control questions (“Now, please check the answer number 2 (number 4)”) or who provided a suspicious combination of answers to other questions (too high/low body height, weight, age, an unrealistically high number of neuropsychiatric disorders, Alzheimers disorder (1 subject) and Parkinson’s disorder (2 subjects) who answered all or nearly all questions by the same code, etc.) were filtered out. We also eliminated all records of subjects who were less than 15 years old, did not answer how red their hair is, or did not answer any questions concerning their health. All semi-continuous variables (sexual desire and nine impaired health syndromes) had a highly asymmetric distribution that cannot be regularized by any mathematical transformation. Therefore, we transformed these variables to ordinal variables 1–10 with manually spaced borders of intervals to get comparable numbers of subjects in particular classes.

The final set contained data from 4,117 subjects. The distribution of all relevant (semi-continuous and ordinal) variables were visually checked and the classes containing less than 3% of subjects were merged with the adjacent class. We also computed a new semi-continuous variable, sexual desire, as Maximum(sexually aroused by opposite sex people, sexually aroused by same sex people). Statistical analysis was performed with the statistical packages IBM SPSS v 21 (logistic regression, ordinal regression, linear regression) and Statistica v. 10.0. (descriptive statistics, Kendall and Spearman correlation tests). To compute partial Kendall Tau and the significance of each variable after controlling for age, we used an Excel spreadsheet available at: http://web.natur.cuni.cz/flegr/programy.php. The correction for multiple tests was done using Benjamini-Hochberg procedure with the false discovery rate pre-set to 0.25^26^.

### Differences between preregistered and realized protocol

In the preregistered protocol, we planned to use darkness of eye and hair, and waviness of hair as negative controls to redness of hair. By a technical error, we used a questionnaire module with the item “darkness of skin” instead of “waviness of hair”. Therefore, we used the darkness of skin (rated on the same Likert scale 0–5 as other variables) instead the hair waviness in our analyses. By a mistake, we also preregistered measurement sexual desire with a 6-point scale; however, this variable was measured with an 100-point scale. Because of the strong asymmetry of all semi-continuous variables, we decided to transform these variables (scales 0–100) to ordinal variables (scales 1–10) and therefore substitute the ANOVA tests with corresponding ordinal regression tests. To compensate for this change in the protocol, we used the more conservative two-sided variant of all statistical tests instead of the preregistered one-sided variant of the tests.

## Results

### Descriptive statistics

The final set contained the data of 2,668 women and 1,449 men who provided information about their hair redness and also at least some data about their health. The mean age of women, 32.9 (s.d. = 12.3), was lower than mean age of men, 35.1 (s.d. = 12.3, t_4115_ = −5.45, p < 0.00005). More details about the structure of the population is shown in Table 1. In all analyses, we always used the ordinal variables (0–5). However, the transformation of the ordinal variable “intensity of redness” to binary variable “rather non-red” (codes 0, 1, 2) and “rather red” (codes 3, 4, 5) suggest that about 10.31% redhead women and 6.63% redhead men existed in the Czech population of mixed Celtic-Slavic ethnicity. The distribution of nearly all variables, both health-related variables and variables describing the pigmentation of eye, hair, and skin, was highly asymmetric; see the Supplementary Figures 1–20. Moreover, nearly all output variables correlated with age, and some also with size of the settlement (Table 2). Therefore, in accordance with the preregistered protocol, we used nonparametric partial Kendall correlation with age as a covariate as the main method for testing the hypotheses related to the semi-continuous variables (the symptoms of impaired mental health and sexual desire).

**Table 1:** Pigmentation of eye, hair, and skin, physical and mental health, and number of children reported by the participants of study.

		The subjects responses					
		0	1	2	3	4	5
Eye darkness	♀	380 (14.27%)	567 (21.29%)	518 (19.45%)	491 (18.44%)	439 (16.49%)	268 (10.06%)
	♂	196 (13.56%)	342 (23.67%)	185 (12.8%)	233 (16.12%)	318 (22.01%)	171 (11.83%)
Hair darkness	♀	36 (1.35%)	317 (11.91%)	769 (28.9%)	775 (29.12%)	645 (24.24%)	119 (4.47%)
	♂	16 (1.11%)	184 (12.73%)	291 (20.14%)	397 (27.47%)	452 (31.28%)	105 (7.27%)
Hair redness	♀	1456 (54.57%)	662 (24.81%)	275 (10.31%)	196 (7.35%)	66 (2.47%)	13 (0.49%)
	♂	1020 (70.39%)	256 (17.67%)	77 (5.31%)	64 (4.42%)	27 (1.86%)	5 (0.35%)
Skin darkness	♀	399 (14.97%)	1029 (38.6%)	732 (27.46%)	445 (16.69%)	58 (2.18%)	3 (0.11%)
	♂	110 (7.61%)	553 (38.24%)	470 (32.5%)	260 (17.98%)	49 (3.39%)	4 (0.28%)
Physical health	♀	18 (1.01%)	69 (3.86%)	263 (14.7%)	521 (29.12%)	648 (36.22%)	270 (15.09%)
	♂	5 (0.52%)	32 (3.36%)	133 (13.96%)	237 (24.87%)	401 (42.08%)	145 (15.22%)
Mental health	♀	43 (2.4%)	130 (7.26%)	287 (16.03%)	488 (27.26%)	563 (31.45%)	279 (15.59%)
	♂	21 (2.21%)	49 (5.15%)	126 (13.25%)	223 (23.45%)	318 (33.44%)	214 (22.5%)
Mental h. drugs	♀	1485 (87.51%)	133 (7.84%)	54 (3.18%)	25 (1.47%)		
	♂	839 (91.79%)	46 (5.03%)	19 (2.08%)	10 (1.09%)		
Other drugs	♀	985 (57.3%)	410 (23.85%)	178 (10.35%)	84 (4.89%)	62 (3.61%)	
	♂	589 (64.09%)	158 (17.19%)	88 (9.58%)	38 (4.13%)	46 (5.01%)	
Number disorders	♀	2156 (80.81%)	306 (11.47%)	106 (3.97%)	100 (3.75%)		
	♂	1216 (83.92%)	141 (9.73%)	53 (3.66%)	39 (2.69%)		
Children	♀	940 (49.47%)	346 (18.21%)	474 (24.95%)	112 (5.89%)	28 (1.47%)	
	♂	433 (44.78%)	169 (17.48%)	258 (26.68%)	85 (8.79%)	22 (2.28%)	

The table shows the number and the percentage of female (♀) and male (♂) subjects providing particular response to the questions on eye darkness, hair darkness etc. (column 1). In the upper part of the table the population is stratified using the scale 0–5 (5 = the highest eye darkness, hair darkness etc.). In the lower part of the table the stratification was done according to the number of different drugs prescribed by a doctor taken in past month (separately for mental health disorders and for other disorders), the number of checked neuropsychiatric disorders in the provided list of 26 disorders, and the number of participant’s biological children. In this part of the table, the highest category, e.g. 4 (children), means 4 and more. For the next statistical analyses, the categories with less than 3% subjects were merged with adjacent categories.

**Table 2:** Effects of age and size of settlements in which responders currently live.

	Age			Size of the place of living		
	N	Spearman R	p-value	N	Spearman R	p-value
Eye darkness	4108	−0.022	0.162	4070	0.020	0.208
Hair darkness	4106	0.013	0.402	4068	0.012	0.435
Hair redness	4117	−0.162	**0**.**000**	4079	0.045	**0**.**004**
Skin darkness	4112	0.141	**0**.**000**	4074	−0.047	**0**.**003**
No. children	2867	0.705	**0**.**000**	2840	−0.148	**0**.**000**
Sexual desire	2731	0.072	**0**.**000**	2710	−0.040	**0**.**036**
Physical health	2742	−0.060	**0**.**002**	2721	0.005	0.811
Mental health	2741	0.145	**0**.**000**	2720	−0.022	0.257
Drugs mental health	2611	0.109	**0**.**000**	2593	−0.003	0.893
Drugs other	2638	0.134	**0**.**000**	2620	0.003	0.872
No. disorders	4117	0.040	**0**.**009**	4079	0.028	0.072
Anxieties	2721	−0.166	**0**.**000**	2701	0.045	**0**.**019**
Phobias	2701	−0.207	**0**.**000**	2681	0.020	0.303
Depressions	2705	−0.169	**0**.**000**	2686	0.025	0.201
Manias	2667	−0.155	**0**.**000**	2647	0.019	0.337
Obsessions	2677	−0.255	**0**.**000**	2657	0.022	0.263
Auditory hallucinations	2653	−0.078	**0**.**000**	2633	0.005	0.795
Visual hallucinations	2651	−0.011	0.570	2631	−0.003	0.868
Burnout s.	2702	−0.014	0.473	2682	0.037	0.059
Headache	2710	−0.137	**0**.**000**	2690	0.013	0.506

The table shows population size (N), Spearman correlation coefficients (R) and p value for particular associations of age and size of place of living with the variables listed in the first column. A positive R means a positive association between, for example, physical health (or, e.g., depression) and age or the size of settlement. Significant values are typed in bold and 0.000 denote p < 0.0005.

### Confirmatory section of the study

Three hypotheses were preregistered before the start of present study

1. *Reported intensity of red hair color will correlate positively with probability of reporting physical health problems*.
2. *Reported intensity of red hair color will correlate positively with probability of reporting mental health problems* (*anxiety*, *depression*, *etc*.).
3. *Reported intensity of red hair color will correlate positively with probability of reporting diagnosed mental health disorders*.

**Figure 1:**
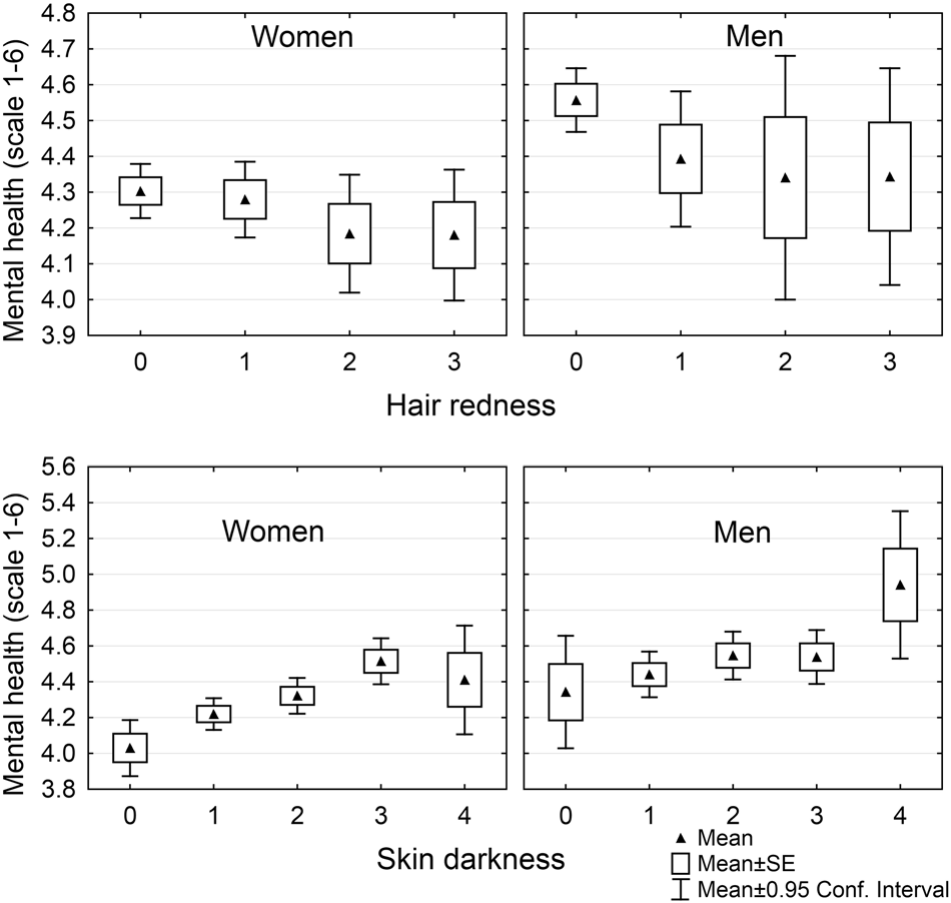
Association between mental health and intensity of pigmentation of hair and skin. Hair redness and skin darkness were self-rated on the scale 0–5; however, the classes containing less than 3% of subjects (4 and 5 for hair redness and 5 for skin darkness) were merged with the adjacent class.

Partial Kendall correlation can be used for an analysis of binary, ordinal, and continuous data, regardless of their distributions. Therefore, all three hypotheses were tested by the same method and all results, after the correction for multiple tests, are shown in Tables 3 and 4. The results suggest that the intensity of hair redness correlated with many physical and mental health-related variables (Table 3) supporting hypotheses 1 and 2, respectively, and also with the incidence of many neuropsychiatric disorders (Table 4). In accordance with hypothesis 3, ten of eleven of significant correlations with neuropsychiatric disorders, except for the correlation with borderline personality disorder in women, were positive. For comparison, the same two tables show the correlation of the health-related variables not only with the focal variable, i.e. with hair redness, but also with hair darkness, eye darkness and skin darkness. Unexpectedly, the associations with skin darkness in women were much stronger and more numerous than with hair redness, see the Fig. 1. This phenomenon was examined in detail in the non-registered part of the study.

**Table 3:** Associations of hair, eye, and skin pigmentation with health-related variables.

	Hair redness		Hair darkness		Eye darkness		Skin darkness	
	Women	Men	Women	Men	Women	Men	Women	Men
No. children	−**0**.055*	−**0**.**048**	0.010	0.032	−0.009	−0.017	**0**.072*	**0**.071*
Sexual desire	−**0**.**029**	−**0**.**031**	0.008	0.005	−**0**.**026**	−0.015	**0**.**029**	**0**.**039**
Physical health	−**0**.**026**	−0.019	**0**.**035**	−0.002	0.014	−0.024	**0**.048*	**0**.061*
Mental health	−0.015	−**0**.**055**	**0**.072*	−0.008	**0**.**036**	0.017	**0**.080*	**0**.**042**
drugs mental health	**0**.**030**	0.008	−**0**.**041**	0.046	−**0**.**032**	0.021	−**0**.086*	−0.011
Drugs other	**0**.**030**	0.028	0.014	−0.032	−0.011	−0.023	−**0**.046*	−**0**.**054**
No. disorders	0.014	**0**.**038**	−0.004	0.023	−**0**.**024**	−0.003	−**0**.075*	−0.012
Anxieties	**0**.068*	**0**.063*	−**0**.**025**	0.033	−**0**.058*	0.011	−**0**.109*	−0.001
Phobias	**0**.045*	**0**.**032**	−0.013	**0**.**056**	−**0**.**028**	**0**.**053**	−**0**.071*	0.007
Depressions	**0**.046*	**0**.067*	−**0**.**038**	0.040	−**0**.**030**	0.016	−**0**.077*	−0.025
Manias	**0**.071*	**0**.**035**	−0.010	−0.003	−0.021	0.004	−**0**.**029**	0.019
Obsessions	**0**.099*	**0**.**055**	−0.004	0.017	−0.010	**0**.**052**	−**0**.**044**	−0.024
Auditory hallucinations	**0**.054*	**0**.067*	−**0**.**028**	−0.036	−**0**.**025**	0.044	0.012	0.001
Visual hallucinations	**0**.058*	**0**.**055**	−**0**.**026**	−0.02	−**0**.**032**	−0.003	−0.009	0.012
Burnout syndromes	**0**.**022**	0.009	−0.020	0.021	−0.004	0.019	−**0**.063*	0.000
Headache	**0**.**044**	−0.021	−0.010	0.016	−0.008	0.014	−**0**.056*	−0.031

The table shows partial Kendall Taus (age controlled). Except the number of children, sexual desire, physical health, and mental health, the positive Taus indicate impaired health. The associations significant after the correction for multiple tests are typed in bold and those with p values less than 0.005 are marked with asterisks.

**Table 4:** Associations of hair, eye, and skin pigmentation with mental health disorders.

	Incidence		Hair redness		Hair darkness		Eye darkness		Skin darkness	
	Women	Men	Women	Men	Women	Men	Women	Men	Women	Men
Phobic disorder	66 (3.73)	35 (3.75)	−0.012	**0**.064*	0.018	**0**.**060**	−0.006	0.004	−**0**.048*	−0.002
Autism spectrum dis.	9 (0.51)	7 (0.75)	**0**.**034**	0.002	0.004	0.015	−0.004	0.032	−**0**.045*	−**0**.**047**
Major depression	120 (6.79)	31 (3.32)	0.019	0.002	−0.011	0.009	−0.008	−0.021	−**0**.053*	−**0**.**045**
Bipolar disorder	20 (1.13)	8 (0.86)	0.005	0.020	−**0**.**042**	−0.022	−0.022	−0.039	−0.038	−0.041
Schizophrenia	1 (0.06)	2 (0.21)	**0**.**040**	−0.023	0.025	0.005	**0**.**033**	**0**.**067**	−0.035	−0.010
General anxiety disorder	99 (5.60)	33 (3.54)	**0**.051*	−0.005	−0.002	**0**.071*	−0.007	−0.009	−**0**.050*	−0.001
Alcohol use disorder	8 (0.45)	16 (1.71)	0.003	0.003	0.012	−0.019	−0.023	−0.029	−0.017	−**0**.**071**
Gambling	1 (0.06)	1 (0.11)	−0.025	**0**.**066**	0.021	0.000	0.023	0.030	−0.001	0.012
Drug use disorder	8 (0.45)	9 (0.96)	0.022	0.019	0.008	0.009	−0.006	0.004	−0.012	−0.035
Posttraumatic stress disorder	51 (2.88)	8 (0.86)	0.010	0.010	−0.026	0.011	−**0**.045*	0.032	−**0**.**031**	−0.019
Obsessive compulsive disorder	33 (1.87)	14 (1.50)	**0**.**030**	**0**.**043**	**0**.**035**	0.029	0.008	0.004	−**0**.**040**	−0.030
Panic disorder	73 (4.13)	27 (2.89)	**0**.**036**	0.022	0.019	−0.024	−0.020	−0.032	−**0**.061*	−0.025
Insomnia primary	29 (1.64)	14 (1.50)	0.014	0.042	−**0**.**041**	−0.013	−0.028	0.033	−**0**.067*	−**0**.067*
Learning disabilities	56 (3.17)	56 (6.00)	−0.002	**0**.067*	0.023	**0**.**045**	0.020	0.000	0.015	0.001
Borderline person. disorder	15 (0.85)	4 (0.43)	−**0**.**029**	−0.039	−**0**.**032**	0.042	0.001	−0.023	−**0**.**043**	−0.026
Antisocial person. disorder	7 (0.40)	4 (0.43)	−0.021	−0.020	0.007	0.035	−0.012	−0.016	0.023	−0.021
Attention deficit hyperactivity dis.	19 (1.07)	19 (2.04)	0.008	0.007	0.006	**0**.076*	−0.009	0.018	−0.013	0.031
Bulimia, anorexia	33 (1.87)	4 (0.43)	−0.001	0.019	−0.016	0.025	−0.008	−0.008	−0.018	−**0**.**050**
Burn−out syndrome	41 (2.32)	32 (3.43)	−0.018	0.029	0.007	−0.005	**0**.**038**	−0.018	−0.030	0.006
Sexual disorder	7 (0.40)	13 (1.39)	−0.024	0.012	0.015	−0.010	0.014	−0.032	0.006	−0.037
Suicide	55 (3.11)	20 (2.14)	0.017	**0**.**049**	−0.008	0.011	−0.004	−0.022	−**0**.**044**	−**0**.070*
Epilepsy	17 (0.96)	14 (1.50)	0.007	0.002	−0.025	0.027	−0.005	0.036	−0.016	0.017
Other disorder	108 (6.11)	29 (3.11)	−0.019	−0.010	**0**.**029**	0.020	0.001	−0.015	−0.012	0.032

The table shows incidences of particular neuropsychiatric disorders (N and percentages) and partial Kendal Taus (age controlled). The positive Tau indicates increased incidence of particular disorder in subjects with red hair, dark hair, dark eye or dark skin. The associations significant after the correction for multiple tests are typed in bold and those with p values less than 0.005 are marked with asterisks. The table shows the incidence of all disorders; however, only the disorders reported by more than nine subjects were taken into consideration in the discussion and the correction for multiple tests.

### Exploratory section of the study

Redness and darkness of hair and darkness of eye were correlated with darkness of skin (hair redness: Tau = −0.141, p < 0.0005; hair darkness: Tau = 0.296, p < 0.0005; eye darkness: Tau = 0.301, p < 0.0005; partial Kendall correlation, age controlled). It is therefore possible that the health-related variables were primarily correlated with skin darkness and only secondarily with hair redness. To test this hypothesis, we studied the association of the health-related variables with hair, eye, and skin pigmentation using more complex general linear models containing all focal (pigmentation-related) variables, age and size of place of living as the independent variables. To avoid problems with asymmetric distributions of semi-continuous variables, we analyzed the transformed 10-point ordinal variables using ordinal, instead of linear regressions; however, the results of ordinal and linear regressions differed only quantitatively. The effects of pigmentation on binary variables were estimated with logistic regression (always with age and size of place of living as covariates). The results for these multivariate analyses are shown in Tables 5, 6 and 7.

**Table 5:** Effects of eye, hair, and skin darkness and hair redness on the ordinal health-related variables.

	All						
	Sex	Place	Age	Eye darkness	Hair darkness	Hair redness	Skin darkness
Children	−0.004	−**0**.054*	**0**.119*	−0.046	**0**.**080**	0.045	**0**.126*
Physical health	**0**.**145**	−0.002	−**0**.011*	−0.040	0.016	−0.036	**0**.133*
Mental health	**0**.282*	−0.002	**0**.019*	−0.002	0.048	−0.026	**0**.111*
Drugs mental	−**0**.540*	0.011	**0**.033*	0.034	0.039	0.080	−**0**.254*
Drugs other	−**0**.**230**	0.017	**0**.033*	−0.003	**0**.**065**	**0**.**098**	−**0**.149*
Disorders	−**0**.**210**	**0**.**028**	**0**.012*	−0.015	**0**.**089**	0.046	−**0**.168*
		**Women**					
		**Place**	**Age**	**Eye darkness**	**Hair darkness**	**Hair redness**	**Skin darkness**
Children		−**0**.062*	**0**.123*	−0.024	0.033	0.046	**0**.**134**
Physical health		0.002	−**0**.**008**	−0.017	0.039	−0.038	**0**.**094**
Mental health		−0.007	**0**.019*	−0.018	**0**.**120**	0.003	**0**.**121**
Drugs mental		0.005	**0**.037*	0.029	−0.052	0.096	−**0**.287*
Drugs other		0.000	**0**.026*	−0.004	0.085	0.082	−**0**.**147**
Disorders		0.022	**0**.018*	−0.007	0.061	0.034	−**0**.228*
		**Men**					
		**Place**	**Age**	**Eye darkness**	**Hair darkness**	**Hair redness**	**Skin darkness**
Children		−**0**.**039**	**0**.113*	−0.085	**0**.**161**	0.055	0.106
Physical health		−0.011	−**0**.018*	−0.078	−0.025	−0.033	**0**.209*
Mental health		0.011	**0**.019*	0.031	−**0**.**097**	−0.118	0.096
Drugs mental		0.025	**0**.**025**	0.032	**0**.**269**	0.090	−0.163
Drugs other		**0**.**057**	**0**.048*	0.005	0.026	0.112	−0.128
Disorders		**0**.**043**	0.000	−0.028	**0**.**133**	0.103	−0.046

The table shows the results (slopes b) of multivariate ordinal regressions analyzing the associations between the health-related variables (first column) and focal variables and covariates (first row). Female sex was coded as 0 and male sex as 1, therefore a positive b means that men scored higher in the particular variable. The associations significant after the correction for multiple tests are typed in bold and those with p values less than 0.005 are marked with asterisks.

**Table 6:** Effects of eye, hair, and skin darkness and hair redness on mental health symptoms.

	All						
	Sex	Place	Age	Eye darkness	Hair darkness	Hair redness	Skin darkness
Sexual desire	**0**.431*	−**0**.**023**	**0**.**006**	−**0**.**083**	0.050	−0.020	0.112
Anxieties	−**0**.509*	0.008	−**0**.**017**	−0.042	**0**.**074**	**0**.121*	−**0**.135*
Phobias	−**0**.**572**	−0.009	−0.027	0.004	0.054	0.037	−**0**.**110**
Depressions	−**0**.233*	0.005	−**0**.021*	−0.008	0.018	**0**.**083**	−**0**.122*
Manias	0.025	0.004	−**0**.024*	−0.025	0.011	**0**.118*	0.011
Obsessions	**0**.**124**	−0.001	−**0**.038*	0.020	0.036	**0**.147*	−**0**.**071**
Auditory hallucination	−0.052	0.000	−**0**.020*	0.006	−**0**.**082**	**0**.**095**	**0**.**074**
Visual hallucination	−**0**.315*	−0.002	−**0**.**009**	−0.049	−0.021	**0**.**100**	0.044
Burnout syndrome	−**0**.**120**	**0**.**019**	−0.002	0.033	0.000	0.014	−**0**.**096**
headache	−**0**.714*	−0.008	−**0**.016*	0.012	0.041	0.027	−**0**.119*
		**Women**					
		**Place**	**Age**	**Eye darkness**	**Hair darkness**	**Hair redness**	**Skin darkness**
Sexual desire		−0.014	0.003	−0.090	0.053	−0.020	**0**.**101**
Anxieties		0.013	−**0**.013*	−0.057	0.052	**0**.**104**	−**0**.203*
Phobias		−0.015	−**0**.028*	−0.020	0.025	0.024	−**0**.150*
Depressions		0.008	−**0**.020*	−0.008	−0.049	0.055	−**0**.**128**
Manias		−0.007	−**0**.022*	−0.034	0.021	**0**.136*	−0.033
Obsessions		−0.005	−**0**.035*	−0.016	0.035	**0**.168*	−0.060
Auditory hallucination		0.003	−**0**.019*	−0.056	−0.046	0.070	**0**.**092**
Visual hallucination		0.004	−**0**.**010**	−0.061	−0.025	**0**.**099**	0.024
Burnout syndrome		0.018	0.002	0.036	−0.004	0.025	−**0**.139*
Headache		−0.008	−**0**.017*	0.011	0.033	0.055	−**0**.**130**
		**Men**					
		**Place**	**Age**	**Eye darkness**	**Hair darkness**	**Hair redness**	**Skin darkness**
Sexual desire		−0.014	0.003	−**0**.**090**	0.053	−0.020	0.101
Anxieties		−0.005	−**0**.022*	−0.024	0.107	**0**.**186**	0.013
Phobias		0.006	−**0**.025*	0.042	0.096	0.085	−0.015
Depressions		−0.005	−**0**.022*	−0.014	**0**.**159**	**0**.**183**	−0.118
Manias		0.026	−**0**.027*	−0.006	−0.030	0.081	0.103
Obsessions		0.005	−**0**.041*	0.079	0.020	0.110	−0.081
Auditory hallucination		−0.005	−**0**.022*	**0**.**109**	−0.142	**0**.**170**	0.056
Visual hallucination		−0.015	−0.006	−0.026	−0.021	0.104	0.100
Burnout syndrome		0.023	−**0**.**009**	0.030	−0.012	−0.008	−0.006
Headache		−0.006	−**0**.**013**	0.016	0.043	−0.044	−0.094

The table shows the results (slopes b) of multivariate ordinal regressions analyzing the associations between the mental health related symptoms (first column) and focal variables and covariates (first row). Female sex was coded as 0, male as 1, therefore the positive b means that men scored higher in the particular variable. The associations significant after the correction for multiple tests are typed in bold and those with p values less than 0.005 are marked with asterisks.

**Table 7:** Effects of hair, eye, and skin pigmentation on incidence of neuropsychiatric disorders.

	N (%)	Sex	Place	Age	Eye darkness	Hair darkness	Hair redness	Skin darkness
Phobic disorder	101 (3.74)	0.092	0.035	−**0**.030*	−0.042	**0**.**266**	−0.015	−**0**.**222**
Autism spectrum disorder	16 (0.59)	0.748	−0.096	−0.025	0.220	0.177	0.297	−**0**.937*
Major depression	151 (5.59)	−**0**.702*	**0**.083*	0.009	0.018	0.021	0.042	−**0**.**231**
Bipolar disorder	28 (1.04)	−0.212	0.054	0.020	−0.044	−0.209	0.070	−**0**.**353**
Schizophrenia	3 (0.11)	2.892	0.282	**0**.**287**	19.162	−0.237	1.581	−**2**.**458**
General anxiety disorder	132 (4.89)	−**0**.**417**	0.044	0.001	−0.041	**0**.**213**	0.126	−**0**.**217**
Alcohol use disorder	24 (0.89)	**1**.430*	0.065	0.017	−0.126	0.173	0.028	−**0**.**520**
Gambling	2 (0.07)	0.855	−0.331	0.004	0.994	−0.364	0.833	0.257
Drug use disorder	17 (0.63)	**0**.**840**	0.084	0.013	0.025	0.372	0.207	−0.353
Posttraumatic stress disorder	59 (2.18)	−**1**.227*	−0.026	0.011	−0.083	−0.005	0.005	−0.179
Obsessive compulsive disorder	47 (1.74)	−0.029	0.017	−**0**.**032**	0.005	**0**.**368**	0.243	−**0**.**340**
Panic disorder	100 (3.7)	−0.304	0.011	0.008	−0.λ079	**0**.**200**	0.126	−**0**.**299**
Insomnia primary	43 (1.59)	0.017	0.001	**0**.**031**	0.179	−0.114	0.203	−**0**.710*
Learning disabilities	112 (4.15)	**0**.829*	0.039	−**0**.055*	−0.040	0.156	0.150	0.069
Borderline personality disorder	19 (0.7)	−0.629	−0.053	−0.015	0.079	−0.014	−0.494	−**0**.**669**
Antisocial personality disorder	11 (0.41)	0.110	0.064	−**0**.**093**	−0.335	0.423	−0.311	0.154
Attention deficit hyperactivity dis.	38 (1.41)	**0**.**845**	0.038	−**0**.067*	−0.129	**0**.**409**	0.243	0.003
Bulimia, anorexia	37 (1.37)	−**1**.**423**	0.010	−0.013	0.045	−0.044	−0.090	−0.232
Burn−out syndrome	73 (2.7)	**0**.**385**	0.016	**0**.**023**	0.107	−0.003	0.061	−0.167
Sexual disorder	20 (0.74)	**1**.**323**	0.112	−0.029	−0.077	0.071	−0.325	−0.202
Suicide	75 (2.78)	−0.313	−0.029	0.012	0.030	0.126	0.132	−**0**.421*
Epilepsy	31 (1.15)	0.463	−0.018	0.002	0.106	−0.094	0.055	−0.023
Other disorder	137 (5.07)	−**0**.803*	0.011	**0**.024*	−0.055	**0**.**189**	−0.065	−0.051

The table shows effects of hair, eye, and skin pigmentation on the incidences of particular neuropsychiatric disorders (slopes b) estimated with logistic regression with the variables listed in the first row as independent factors and covariates. The second column shows the incidence of the disorders (N and percentages). Positive b means that the men, subjects living in larger cities, older subjects or subjects with more intensive pigmentation have a higher incidence of the particular disorder. The associations significant after the correction for multiple tests are typed in bold and those with p values less than 0.005 are marked with asterisks.

## Discussion

In accordance with the preregistered hypotheses and already published data, the intensity of redness of hair negatively correlated with subjectively rated physical and mental health, and positively with number of different drugs prescribed by doctors for mental health problems and for other problems that the participants were taking during the past month. However, a serendipitous discovery achieved due to using the wrong questionnaire module with the question on skin darkness instead on hair waviness, and the follow-up analyses, showed that low intensity of skin pigmentation, rather than hair redness, is most likely responsible for the observed and reported associations between hair redness and various health problems.

This immediately suggests two possible mechanisms that could be responsible for the observed and reported associations between hair and skin pigmentation and physical, and especially mental, health – namely folic acid deficiency, the possible result of photolysis of folic acids in subjects with pale skin, and vitamin D deficiency, the possible result of inadequate photosynthesis of provitamin D in subjects with sun-sensitive skin who avoid sunbathing.

Folic acid is a part of important cofactors of many enzymes participating in the synthesis of nucleic acids and proteins^27^. The presence of these cofactors in high concentration is especially important in rapidly proliferating cells, for example in developing embryos and in germinal tissues. Folic acid and its conjugative form folate deficiency, could result in macrocystic megaloblastic anemia, as well as in specific malformacies in developing embryo, most importantly in various neural tube defects. The folate deficiency in early childhood could also result in growth retardation and haematologic defects^28^ and decline of cell-mediated immunity^29^. It is also highly probable that due to its role in spermiogenesis, folate deficiency could negatively affect also human male fertility^27,30^. Folic acid concentration in humans is affected by dietary intake (fresh fruits and vegetable, eggs^31^, and by destructive exogenous factors such as excessive ethanol intake and photolytic ultraviolet (UV) radiation^27,32^. Our dark-furry ape ancestors had pale skin on most parts of their bodies. It is believed, that protection of folate from photolysis was (and is) the main selective factor responsible for evolution and sustaining of black melanized skin in our naked species in tropical regions^27^. Theoretically, our pale skin-participants could suffer more often from folate deficiency than the subjects with darker skin. It must be admitted, however, that the clinical spectrum of folate deficiency-related disorders differed from the spectrum of disorders observed in or pale-skin participants (especially psychiatric disorders) and that the folic acid deficiency is relatively rare in non-gravid Czech people. Moreover, the modern vitamin D-folate hypothesis of human skin pigmentation^33^ suggests that two complementary but distinct clines of skin pigmentation resulted from two different selection pressures, the protection against photolysis of folate by >320 nm UV in tropical regions, and the facilitation of vitamin D photosynthesis by ultraviolet light B (UVB) at high latitudes, e.g. in northern and central parts of Europe^27^.

The second possible source of problems of pale skin individuals, including redhead individuals, is vitamin D deficiency. In humans, most vitamin D is produced from 7-dehydrocholesterol photochemically in the epidermis by ultraviolet light B (UVB) as the vitamin D_3_ (cholecalciferol) and rest is obtained from diet as cholecalciferol or ergocalciferol, depending on the composition of food and the amount of UVB radiation at a particular place and time^34^. The main biologically active forms of vitamin D, the 1,25-dihydroxyvitamin D_3_ and the 1,25-dihydroxyvitamin D_2_, are produced by two separate hydroxylations, first in the liver (25-) and then, primarily, in the kidney (1-). However, these hydroxylations also occur in the brain tissue, suggesting, together with the presence of vitamin D receptors on neurons and glial cells, the existence of paracrine functions of vitamin D in many parts of the CNS^35,36^. Numerous biologically active products of an alternative pathway of vitamin D_3_ metabolism are being synthetized in various tissues of mammal body^37–39^. Thirty years ago, vitamin D was supposed to play an important role in calcium metabolism and its insufficiency was discussed mainly in the context of rickets and osteomalacia. Later, however, principal roles of vitamin D in immunity and especially autoimmunity^40^, including type 1 and type 2 diabetes^34,41^ and multiple sclerosis^42,43^ were recognized. In the present time, its key roles in neurophysiology and neuropathology are also intensively studied. Meta-analytic studies showed that vitamin D deficiency plays an important role in the etiology of depression^44^, autism^40,45,46^, Parkinson’s disease^47–49^ and also in some psychoses, including schizophrenia^50,51^. Vitamin D also plays an important role in the regulation of the synthesis of serotonin in the brain and in peripheral organs. Results of many studies currently suggest that vitamin D exerts many effects including trophic functions related to neuronal differentiation and maturation and also plays an important role in neuroprotection^52–54^.

The pale skin of people living out of Africa is considered to be an evolutionary adaptation for living in higher latitudes where the amount of sun shining is low for efficient synthesis of vitamin D for the largest part of the year^55,56^. It is widely believed that in the past, subjects with dark skin had suffered from vitamin D deficiency and therefore had been the targets of negative natural selection^57^. In America, there is a strong negative association between concentration of vitamin D and skin darkness because the subjects of Hispanic, and especially African, origin have a lower concentration of vitamin D than Caucasians. For example, the large study based on the data from the national health and nutrition examination survey showed that the serum 25-hydroxyvitamin D levels were 14.6, 19.9 and 25.7 ng/ml in Blacks, Hispanics, and Whites, respectively^58^. However, within groups of people of the same ethnicity, the relation between the vitamin D level and the skin pigmentation, namely the concentration of eumelanin and pheomelanin, is non-trivial; for review see^59,60^. The skin paleness of native inhabitants of Central Europe primarily reflects not the genetic predisposition for low pigmentation but rather how much time they spend in the sun and how intensively they protect their skin against sunburn by mechanical means (by dress, hats, parasols, shelters) or (probably less effectively^58,61,62^) by suntan lotions. Therefore, paradoxically, the pale skin inhabitants of Czechia (which lies mostly between latitudes 48° and 51°N) have on average less, not more, vitamin D than the dark skin subjects. They either spend less time in direct sun and travel less often to low latitude and high altitude countries, or avoid burning by mechanic and chemical means. This effect, namely the lower concentration of vitamin D in subjects pale skin, was already demonstrated in the famous Australian study^61^. Due to a negative correlation between the intensity of the hair redness and natural skin darkness, red headed people, especially red headed women, protect themselves more aggressively against sunburn, and by doing so, they increase the risk of vitamin D deficiency and associated medical problems. Pale skin also correlates with blond hair. However, blond hair is not a good indication of low synthesis of vitamin D as the darkness of hair (in contrast to darkness of skin) decreases, instead of increases, with the intensity of sunbathing. This could be the reason why the health problems were more strongly associated with hair redness than with hair fairness and possibly also for the positive association between hair darkness and health problems observed in the male participants of our study.

The association with skin pigmentation was stronger and more numerous than with hair redness especially in women. We hypothesize that men, in contrast to women, have rather inaccurate information about self skin pigmentation but accurate enough information about their hair redness. Moreover, we suppose that it is not the pigmentation of skin alone but the chemical, mechanical or the behavioral self-protection against sun-burning in red headed people (and especially in more careful women) that is directly responsible for the observed health problems. Many red headed men with relatively sensitive skin spend many hours in the sun without using strong suntan lotion and this makes their skin darker (and hair lighter) and, in parallel, protects them against negative effects of low concentration of vitamin D.

In general, our data confirmed the results of the previous study on negative effects of hair redness on human health. Still, we observed one very important difference between the already published study and current study. While red headed subjects, especially women, were observed to have more children in the previous study (despite reporting fertility problems more often), our data showed an opposite pattern: namely, the negative correlation of number of biological children with hair redness (and positive correlation with skin darkness). We carefully checked the data and the questionnaires for possible reasons for this discrepancy. The data sets slightly differed by mean age of participants (about 1.5 year younger subjects participated in the present study) and especially by “framing”. The first study was advertised as a study on effects of Rhesus blood type and other biological factors on human performance and health, while the new study was advertised as a project investigating philosophical problems of concept of self, testing evolutionary hypotheses concerning beauty of flowers, and studying moral dilemmas concerning autonomous cars. It is therefore possible that, primarily, subjects with some health problems or with an interest in their health and lifestyle participated in the previous study and that in such a population, the relation between hair and skin pigmentation and fecundity could be different than it is in the population interested in theoretical problems of philosophy, aesthetics and ethics.

Some effects of hair redness probably exist independently of skin pigmentation. For example, the intensity of hair redness, rather than skin darkness, correlated with mania and obsessions in women and with depression, anxieties and auditory hallucinations in men. It is probable that some factors of social environment, e.g. prejudices against red headed subjects, especially red haired men, and higher sexual attractiveness and activity/desire of red headed women and lower sexual attractiveness and sexual activity or desire of red headed men^20,21,63,64^ and a higher risk of bullying victimization in both sexes^21^ could play an independent role in the development of specific syndromes of impaired mental health. Similarly, we observed specific health problems associated with dark hair when the effects of dark skin and red hair were controlled. For example, subjects with dark hair reported more diagnoses of attention deficit hyperactivity disorder, phobic disorder, obsessive-compulsive disorder, anxiety disorder, and panic disorder. Dark-haired men, but not women, reported worse mental health, more drugs for mental health consumed in the past two months and suffering more from depression. As we suggested earlier, these negative effects of dark hair in men could be explained by the existence of negative correlation between hair darkness and time spent in the sun (and therefore the level of vitamin D). In contrast, minimum effects of eye darkness on mental and physical health were observed when other potential confounders were controlled, despite the fact the eye darkness has been reported to correlate with certain personality traits, dominance and trustfulness^65,66^.

The main strength of this study was that its hypotheses and the concrete methods of data analysis had been preregistered before collecting the data, which protect against common cherry picking and p-value fishing artefacts. The disadvantage of the preregistration was that we had to terminate the study 31.12. 2017, despite the fact that the number of participants was less than 30% of what we expected before we started the study. The number was still large enough for reliable analysis of ordinal and semi-continuous variables, however, it was probably not large enough for the reliable analysis of incidence of some rarer neuropsychiatric disorders, such as schizophrenia, Parkinson’s disease and gambling. The main problem of the present study was that the participants self-reported their skin, hair, and eye pigmentation data as well as their health and mental health-related variables, including the incidence of neuropsychiatric disorders. The participants were asked (with an exclamation mark!) to report only such neuropsychiatric disorders that had been diagnosed by a medical doctor. It is, however, possible that at least some of them provided inaccurate information about their mental health. Similarly, the subjects were asked to rate their “natural darkness of skin”. However, some participants probably understand this term as the darkness of skin without any sunbathing and some after sunbathing. It has been shown, however, that stochastic noise can increase only the risk of false negative results of studies (failure to detect an existing association) not false positive results of studies (detecting non-existent associations)^67^. More specific questions related to the darkness of skin, and preferably also some objective empirical data on skin reflectance, should be used in future studies. Similarly, we hypothesized that vitamin D deficiency played a key role in the observed associations. It must be stressed, however, that we have no empirical data concerning the concentration of vitamin D in the participants of the present study (Note: Before the final version of the present paper was accepted for the publication, another preregistered case control study https://osf.io/p23dk on 200 subjects (30% red-headed participants) was finished. The preliminary analysis of data shows that the dark-skinned Czech people have indeed a significantly lower concentration of vitamin D (and the same concentration of folic acid) as the pale-skinned people). Moreover, only rare observations suggesting a lower concentration of vitamin D in subjects with type 1 skin (burn only) exist in literature^61^. In humans, the skin is a very large and very complex organ with numerous endocrine and metabolic functions and some of these functions are directly or indirectly influenced by the UVB radiation. Namely, synthesis and degradation of many molecules that are biologically active on both local and central level, not only the widely discussed synthesis of vitamin D and degradation of folic acids, but also metabolism of melatonin, PTH-related protein, catecholamines, acetylcholine, and many others can be affected, or can affect, darkness of skin and mental and physical health, for review, see^68–72^. On the central level, UVB radiation can activate the hypothalamic-pituitary-adrenal axis, which could strongly influence many facets of physiology, including the activity of immune system^73,74^. Therefore, our folic acid- and vitamin D-based models must only be considered as a working hypotheses until folic acid or vitamin D deficiency in redheads were to be demonstrated by laboratory tests.

## Conclusions

The results of our preregistered study confirmed the existence of associations between hair redness and certain mental health problems. At the same time, it showed that many of these problems primarily depend on the intensity of skin pigmentation or, possibly, on behavior dependent on skin pigmentation, rather than on hair redness. We have suggested that the possible source of the observed health problems is either photolysis-related folic acid deficiency or vitamin D deficiency, and that latter could be related to the avoidance of sun by the subjects with sensitive, pale skin (who frequently also have red hair). It must be stressed, however, that no direct (biochemical) evidence for the lower concentration of vitamin D or folic acid in redheads is available at the moment of our study’s submission.

Our study has both theoretical and practical implications. It shows that particular phenotypical traits should not be studied separately using simple univariate models but always in their interrelation by using complex multivariate models. For such analyses much larger data sets than for univariate analyses are usually necessary to avoid the problems of over-parametrization and detection of non-existing effects. The practical implication of the present study is that the intensity of pigmentation probably plays a surprisingly large role in the etiology of many neuropsychiatric disorders and should be taken into consideration when making diagnoses and estimating prognosis, and possibly also in choosing the method of therapy. The most general message of our study is that the preregistration of studies and their hypotheses could help us to avoid common experimental artefacts; however, the most important answers to relevant biological questions sometimes depends on the explorative part of a study.

## Supplementary information

Supplementary figures 1-20

## Data Availability

The datasets generated during and/or analysed during the current study are available in the figshare repository, https://figshare.com/s/62916a77fbd097c28cb3.
