# Supplementary material for: Skin fairness is a better predictor for impaired physical and mental health than hair redness
Source: Sci Rep. 2019 Dec 2;9:18138. doi: 10.1038/s41598-019-54662-5 (PMC6888829; doi:10.1038/s41598-019-54662-5)
Supplement: Supplementary file 1 — Supplementary figures 1-20 [file 41598_2019_54662_MOESM1_ESM.pdf]

Supplementary material for the paper:

Skin fairness is a better predictor for impaired physical and mental health than hair redness

Jaroslav Flegr & Kateřina Sýkorová

Supplementary figures 1-20: Histograms of analysed variables

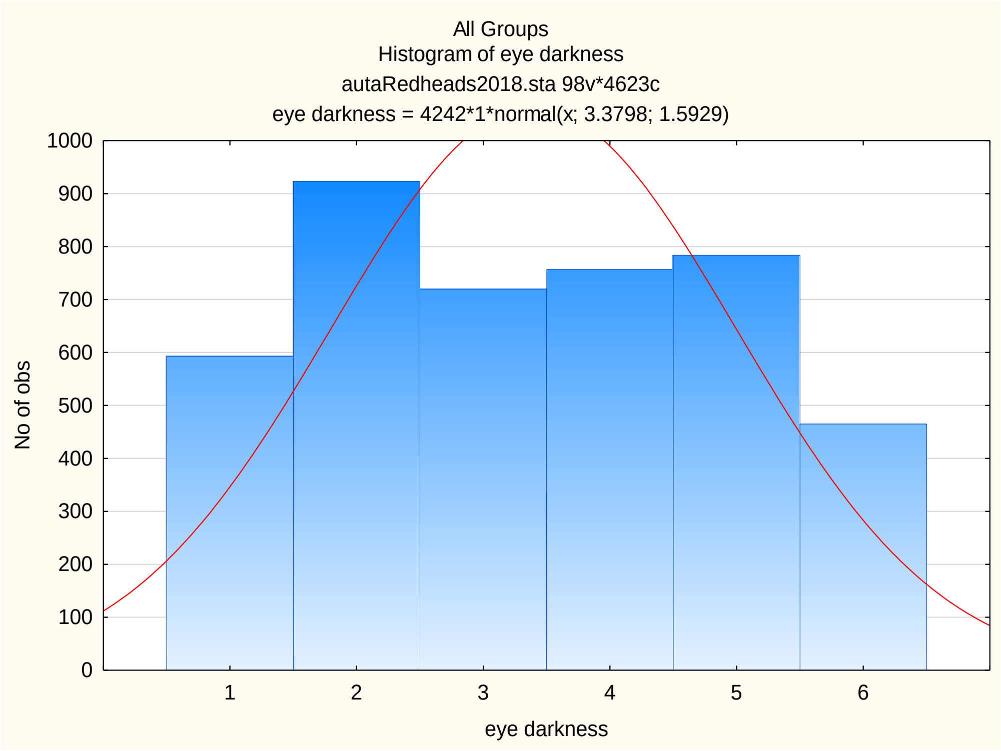
All Groups
Histogram of eye darkness
autaRedheads2018.sta 98v*4623c
eye darkness = 4242*1*normal(x; 3.3798; 1.5929)
No of obs
1000
900
800
700
600
500
400
300
200
100
0
1
2
3
4
5
6
eye darkness

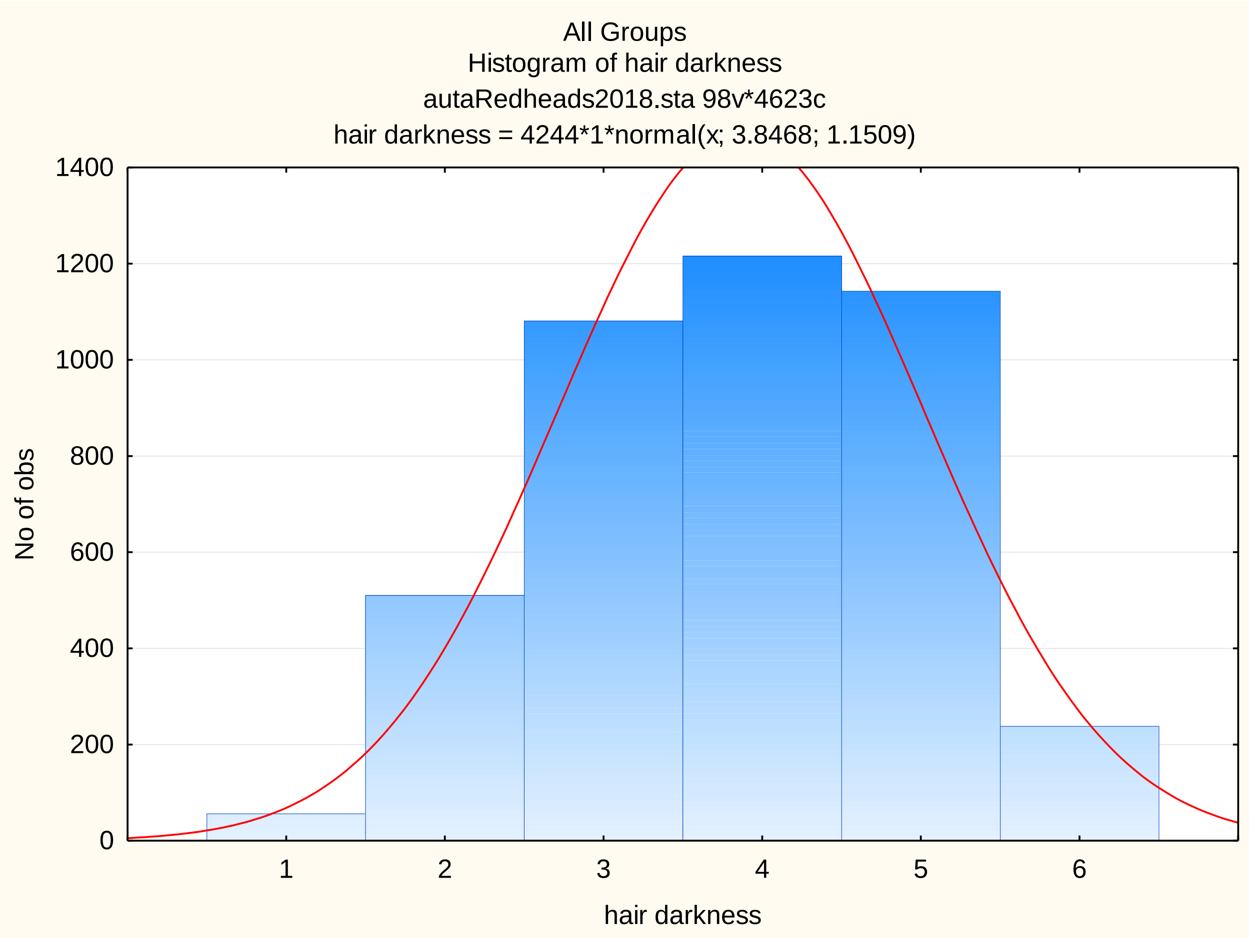
All Groups
Histogram of hair darkness
autaRedheads2018.sta 98v*4623c
hair darkness = 4244*1*normal(x; 3.8468; 1.1509)
No of obs
0
200
400
600
800
1000
1200
1400
1
2
3
4
5
6
hair darkness

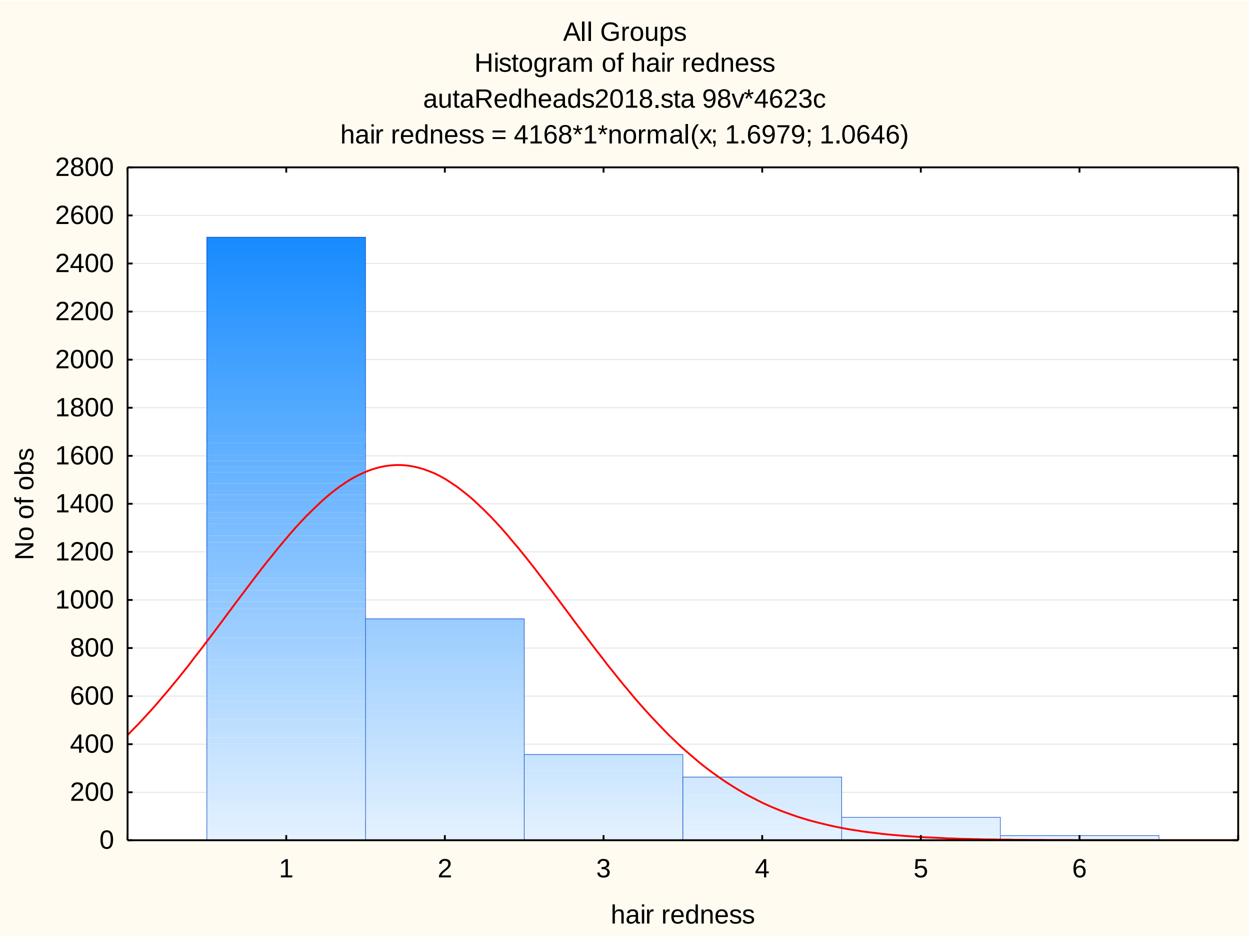
All Groups
Histogram of hair redness
autaRedheads2018.sta 98v*4623c
hair redness = 4168*1*normal(x; 1.6979; 1.0646)
No of obs
0
200
400
600
800
1000
1200
1400
1600
1800
2000
2200
2400
2600
2800
1
2
3
4
5
6
hair redness

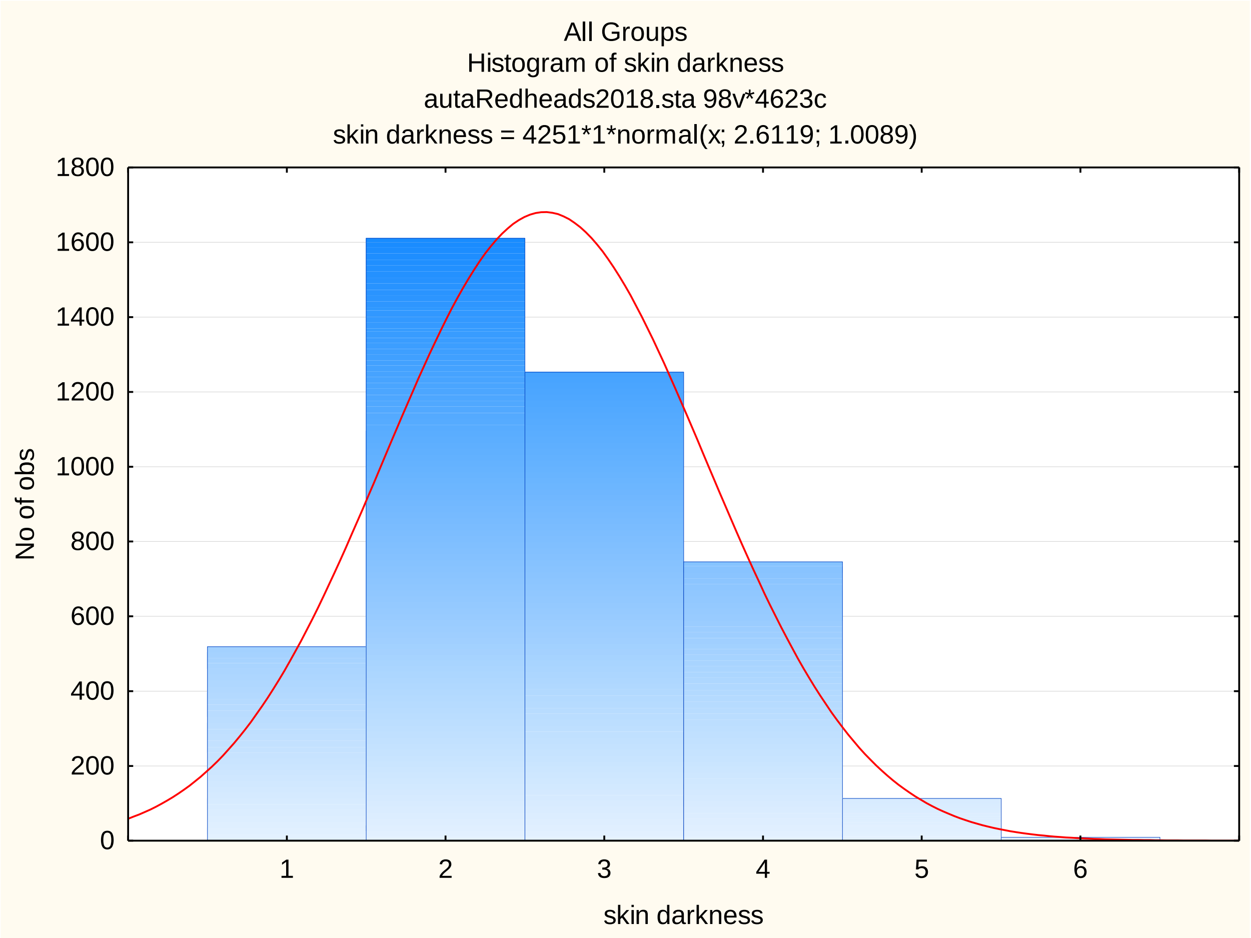

All Groups
Histogram of skin darkness
autaRedheads2018.sta 98v*4623c
skin darkness = 4251*1*normal(x; 2.6119; 1.0089)
No of obs
0
200
400
600
800
1000
1200
1400
1600
1800
1
2
3
4
5
6
skin darkness

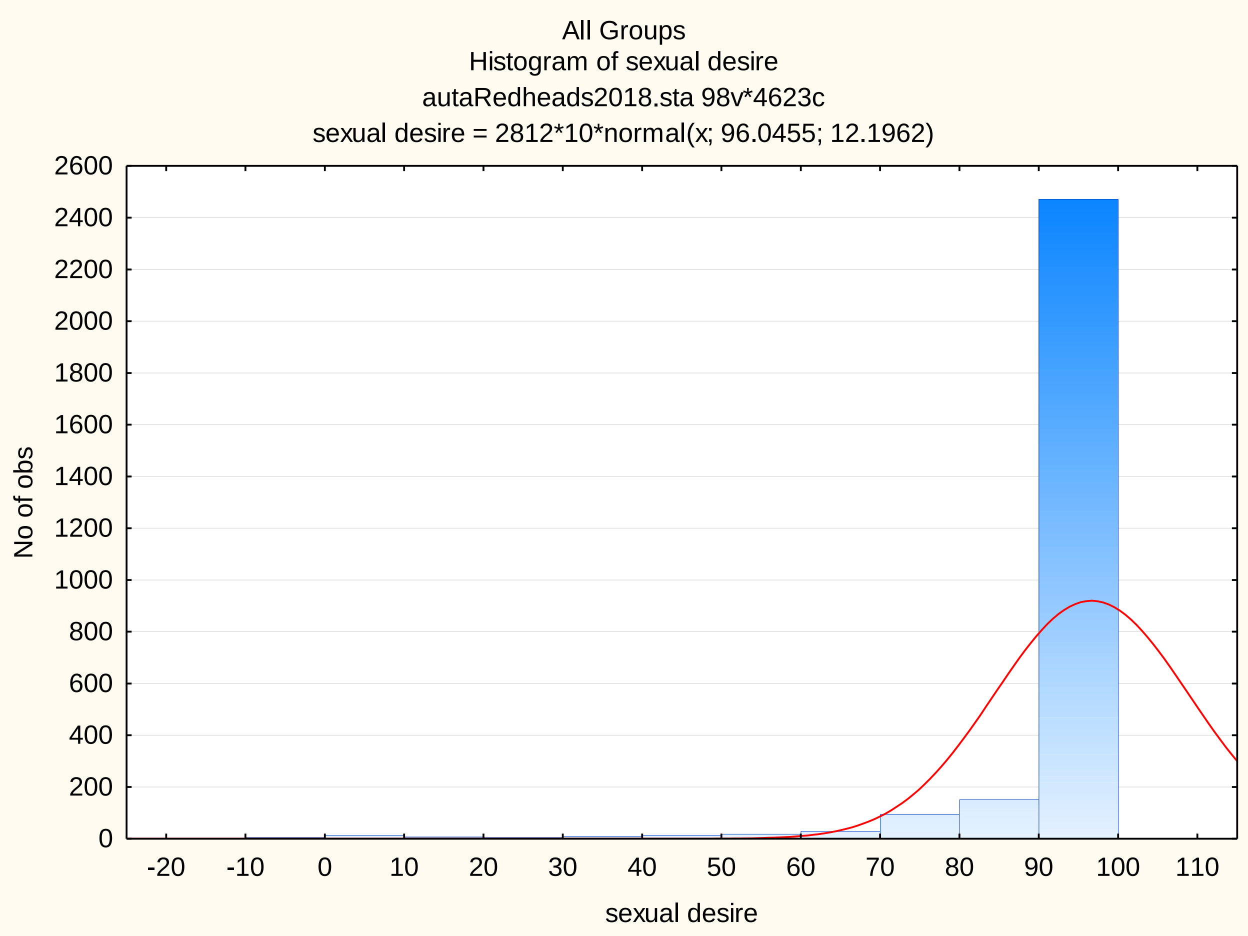
All Groups
Histogram of sexual desire
autaRedheads2018.sta 98v*4623c
sexual desire = 2812*10*normal(x; 96.0455; 12.1962)
No of obs
2600
2400
2200
2000
1800
1600
1400
1200
1000
800
600
400
200
0
-20
-10
0
10
20
30
40
50
60
70
80
90
100
110
sexual desire

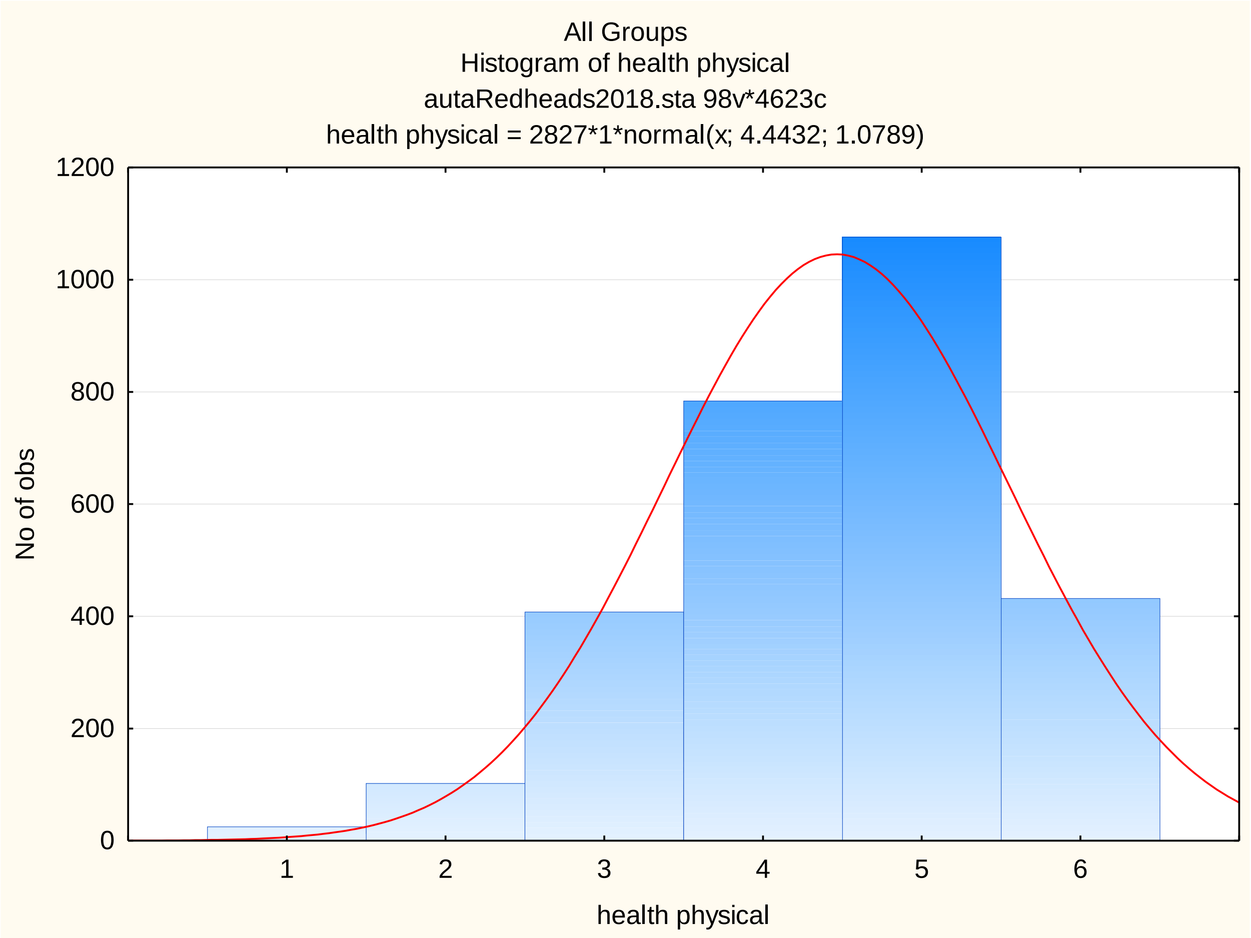
All Groups
Histogram of health physical
autaRedheads2018.sta 98v*4623c
health physical = 2827*1*normal(x; 4.4432; 1.0789)
No of obs
0
200
400
600
800
1000
1200
1
2
3
4
5
6
health physical

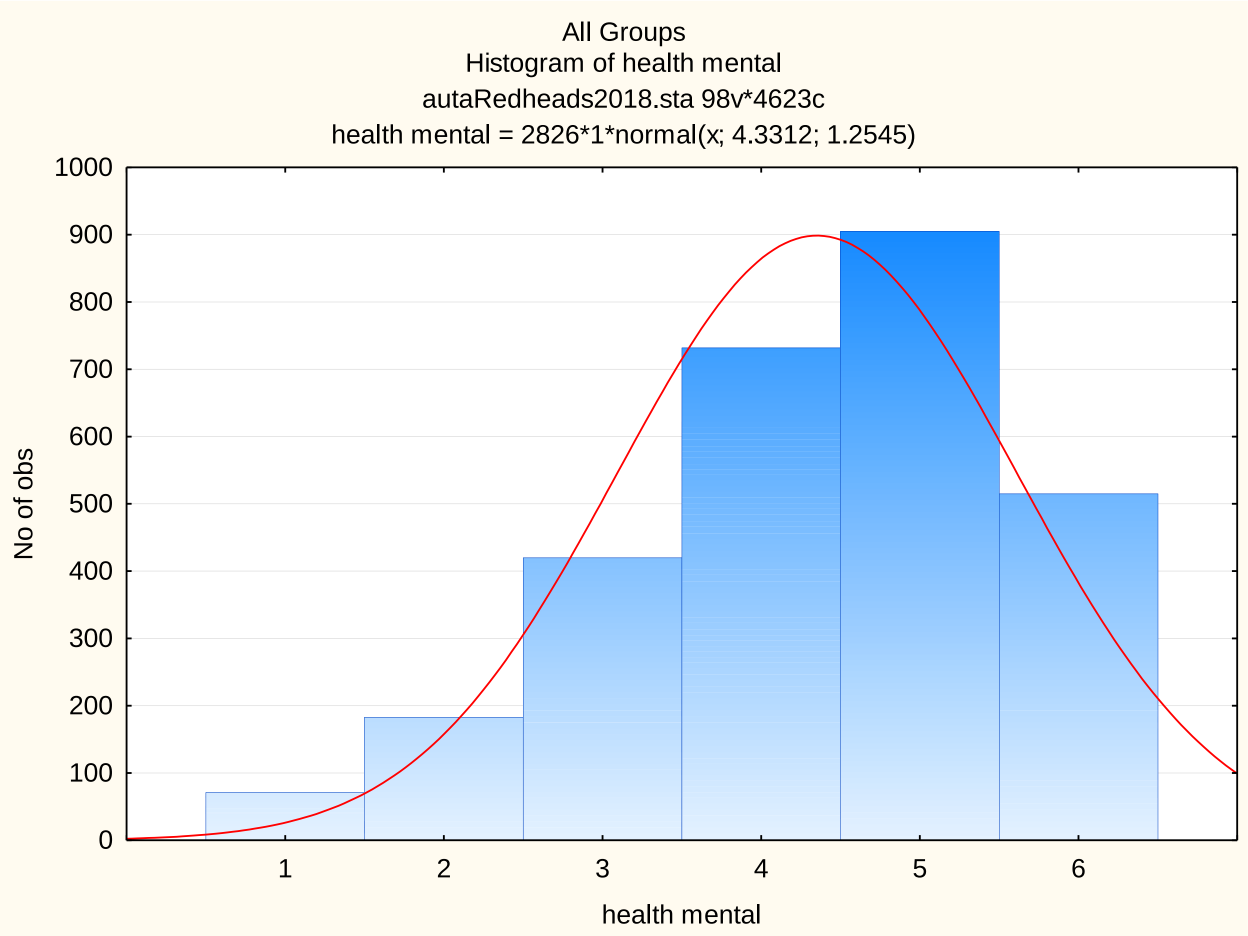

All Groups
Histogram of health mental
autaRedheads2018.sta 98v*4623c
health mental = 2826*1*normal(x; 4.3312; 1.2545)
No of obs
0
100
200
300
400
500
600
700
800
900
1000
1
2
3
4
5
6
health mental

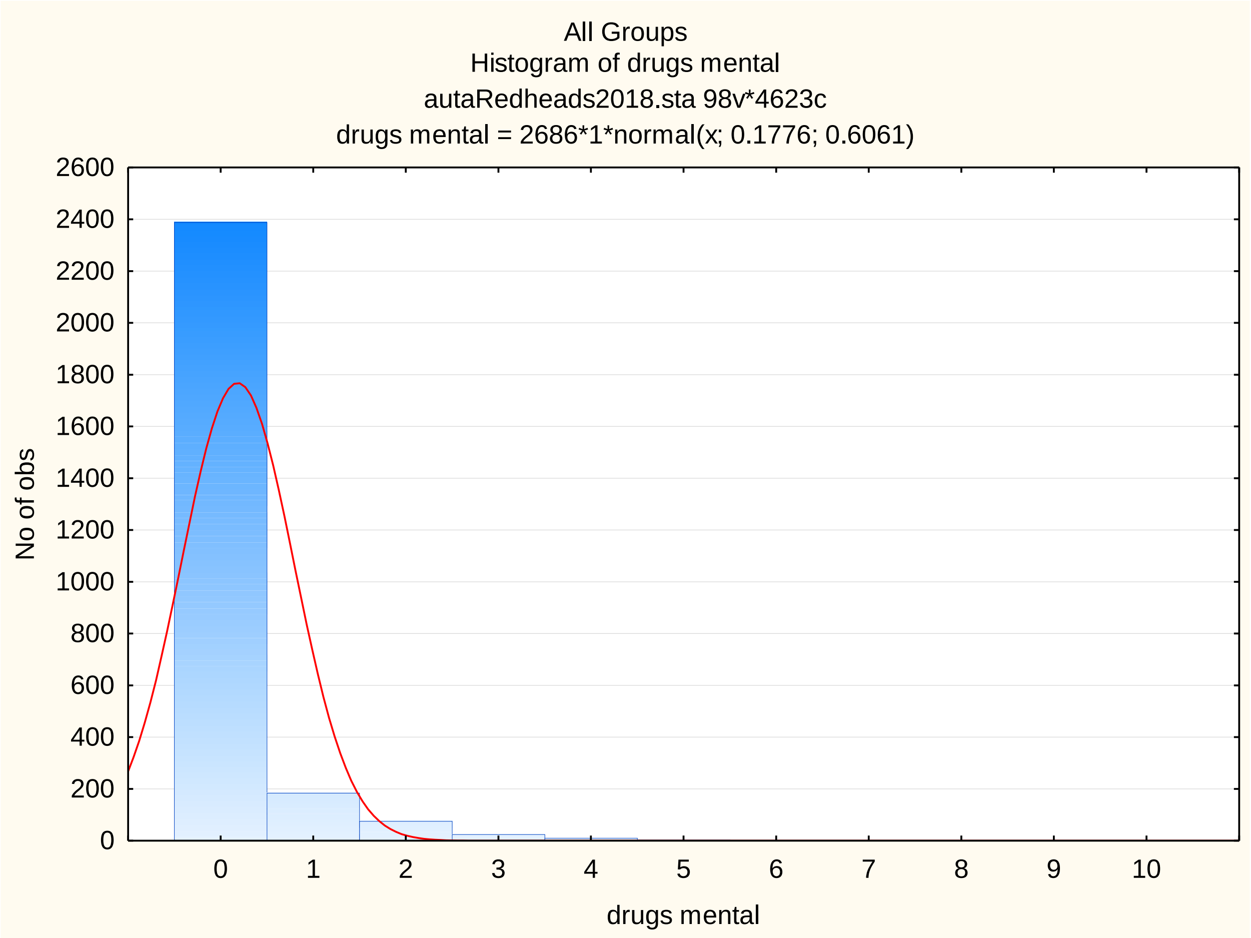
All Groups
Histogram of drugs mental
autaRedheads2018.sta 98v*4623c
drugs mental = 2686*1*normal(x; 0.1776; 0.6061)
No of obs
2600
2400
2200
2000
1800
1600
1400
1200
1000
800
600
400
200
0
0
1
2
3
4
5
6
7
8
9
10
drugs mental

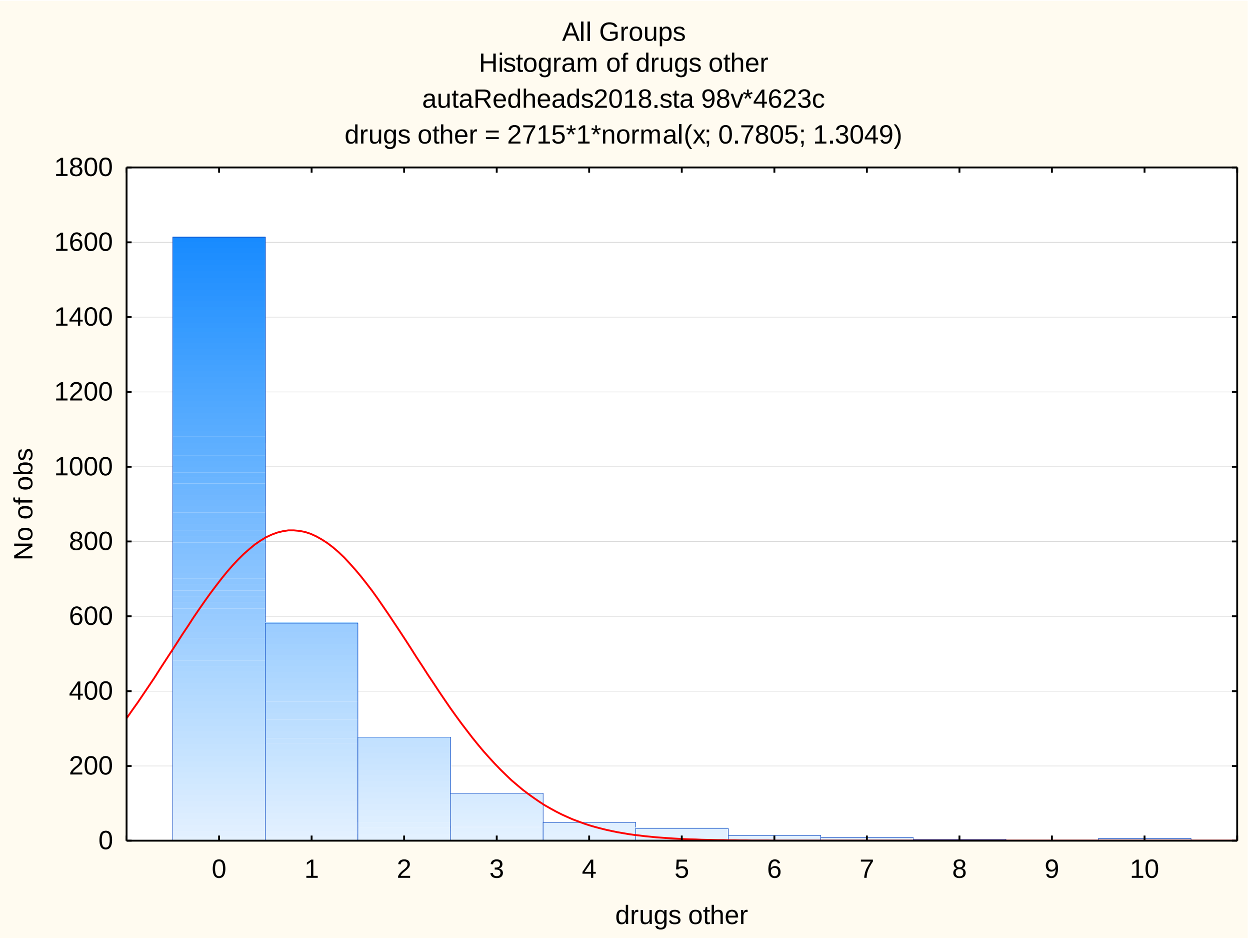
All Groups
Histogram of drugs other
autaRedheads2018.sta 98v*4623c
drugs other = 2715*1*normal(x; 0.7805; 1.3049)
No of obs
0
200
400
600
800
1000
1200
1400
1600
1800
0 1 2 3 4 5 6 7 8 9 10
drugs other

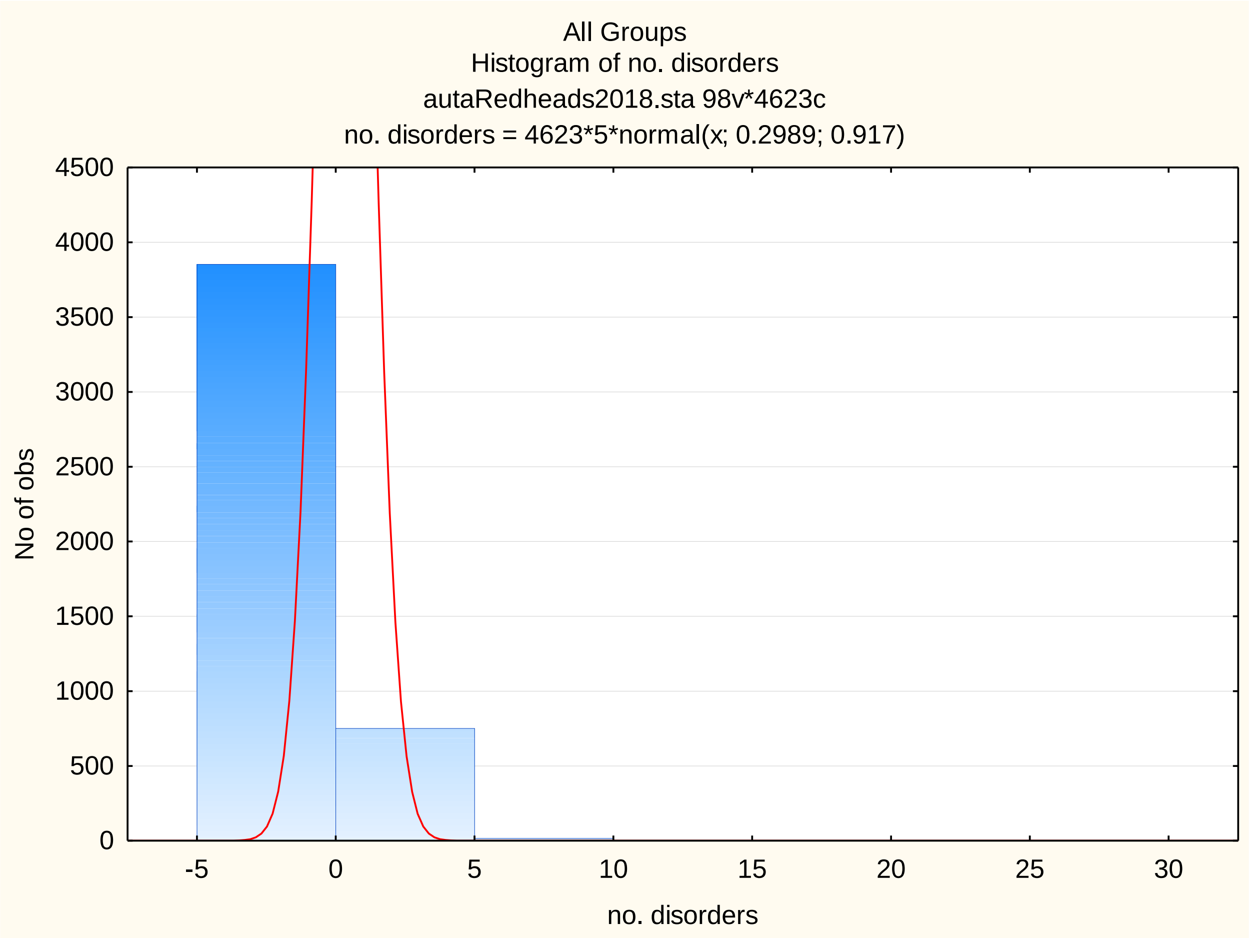
All Groups
Histogram of no. disorders
autaRedheads2018.sta 98v*4623c
no. disorders = 4623*5*normal(x; 0.2989; 0.917)
4500
4000
3500
3000
2500
2000
1500
1000
500
0
No of obs
-5
0
5
10
15
20
25
30
no. disorders

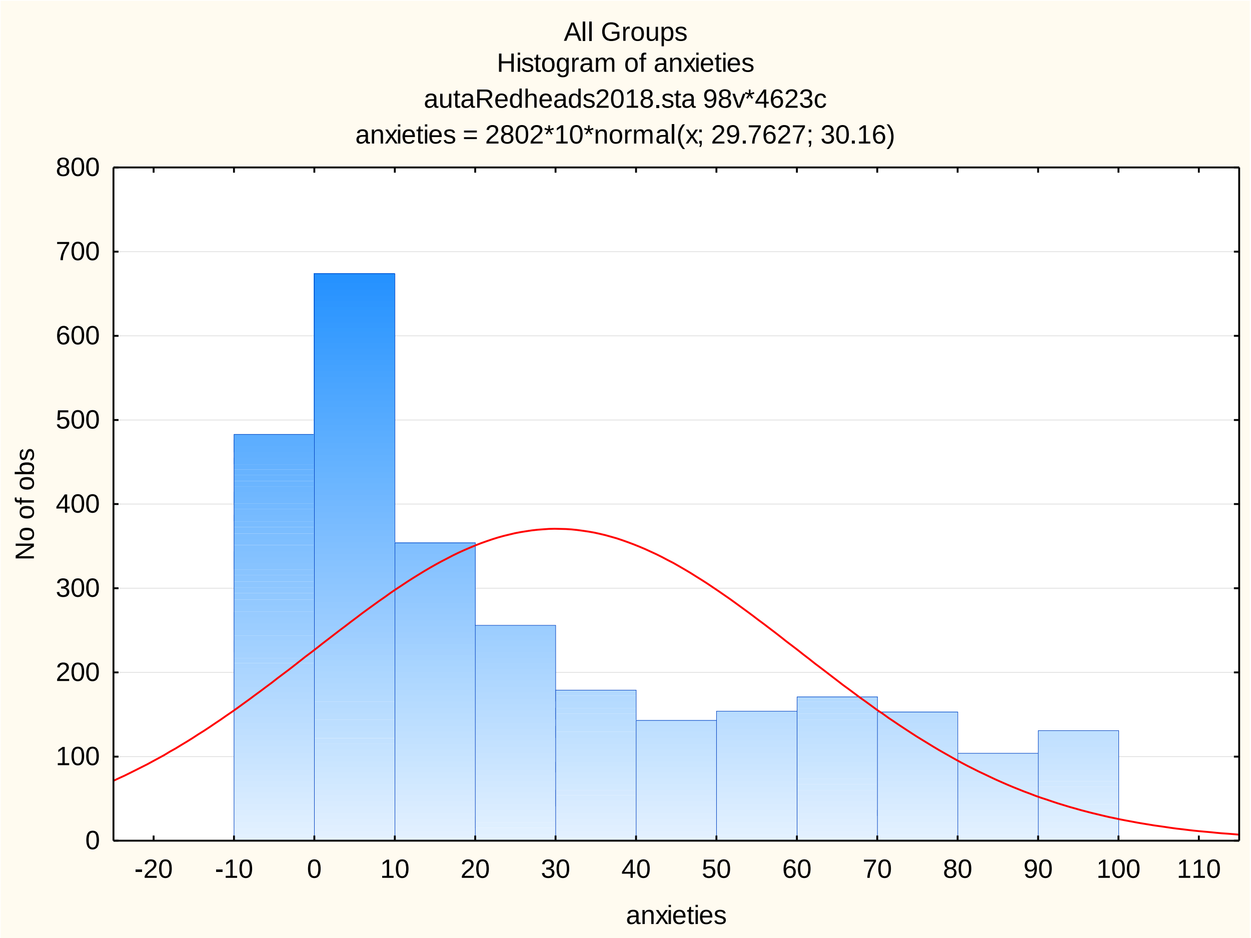

All Groups
Histogram of anxieties
autaRedheads2018.sta 98v*4623c
anxieties = 2802*10*normal(x; 29.7627; 30.16)
No of obs
0
100
200
300
400
500
600
700
800
-20
-10
0
10
20
30
40
50
60
70
80
90
100
110
anxieties

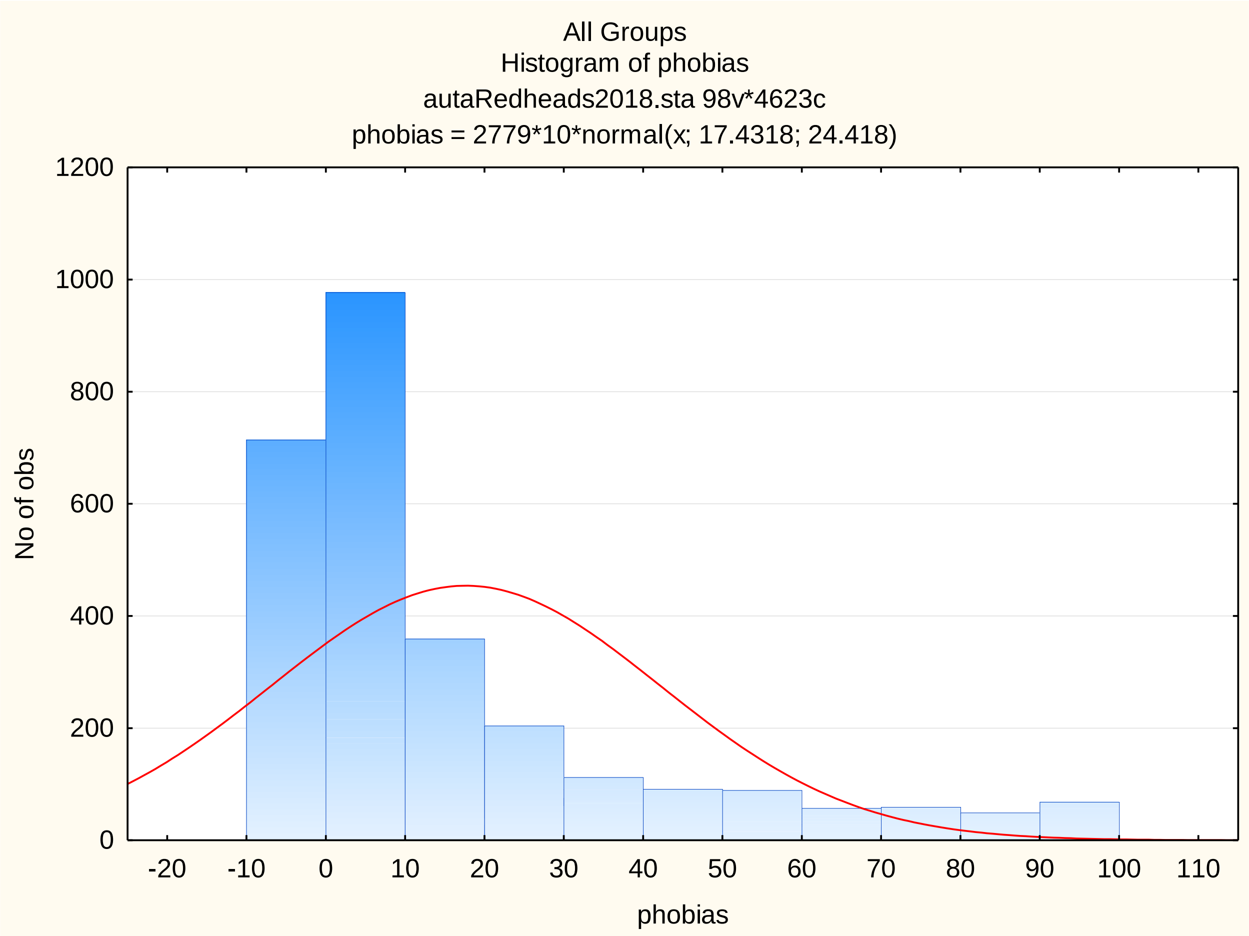
All Groups
Histogram of phobias
autaRedheads2018.sta 98v*4623c
phobias = 2779*10*normal(x; 17.4318; 24.418)
No of obs
0
200
400
600
800
1000
1200
-20
-10
0
10
20
30
40
50
60
70
80
90
100
110
phobias

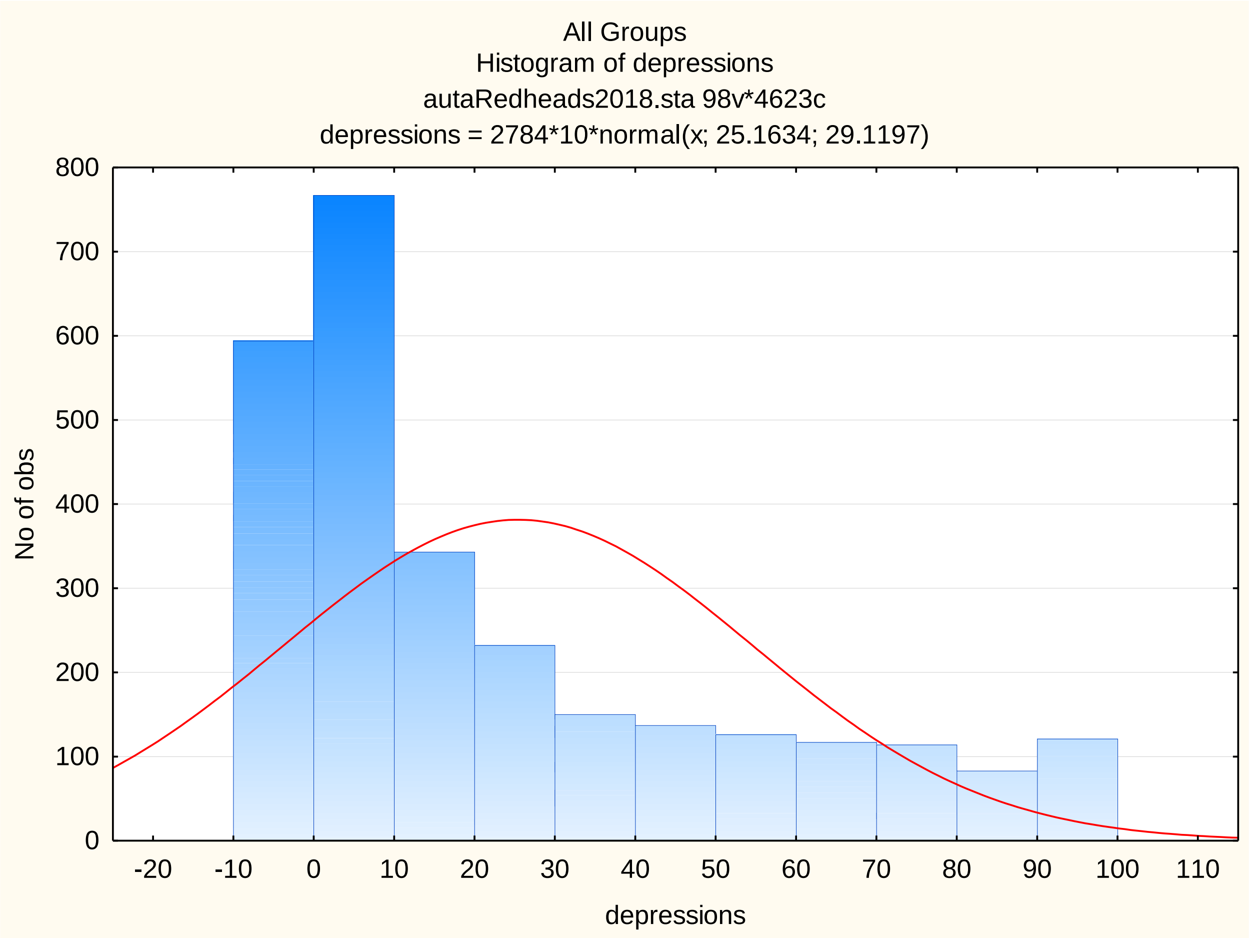
All Groups
Histogram of depressions
autaRedheads2018.sta 98v*4623c
depressions = 2784*10*normal(x; 25.1634; 29.1197)
No of obs
0
100
200
300
400
500
600
700
800
-20
-10
0
10
20
30
40
50
60
70
80
90
100
110
depressions

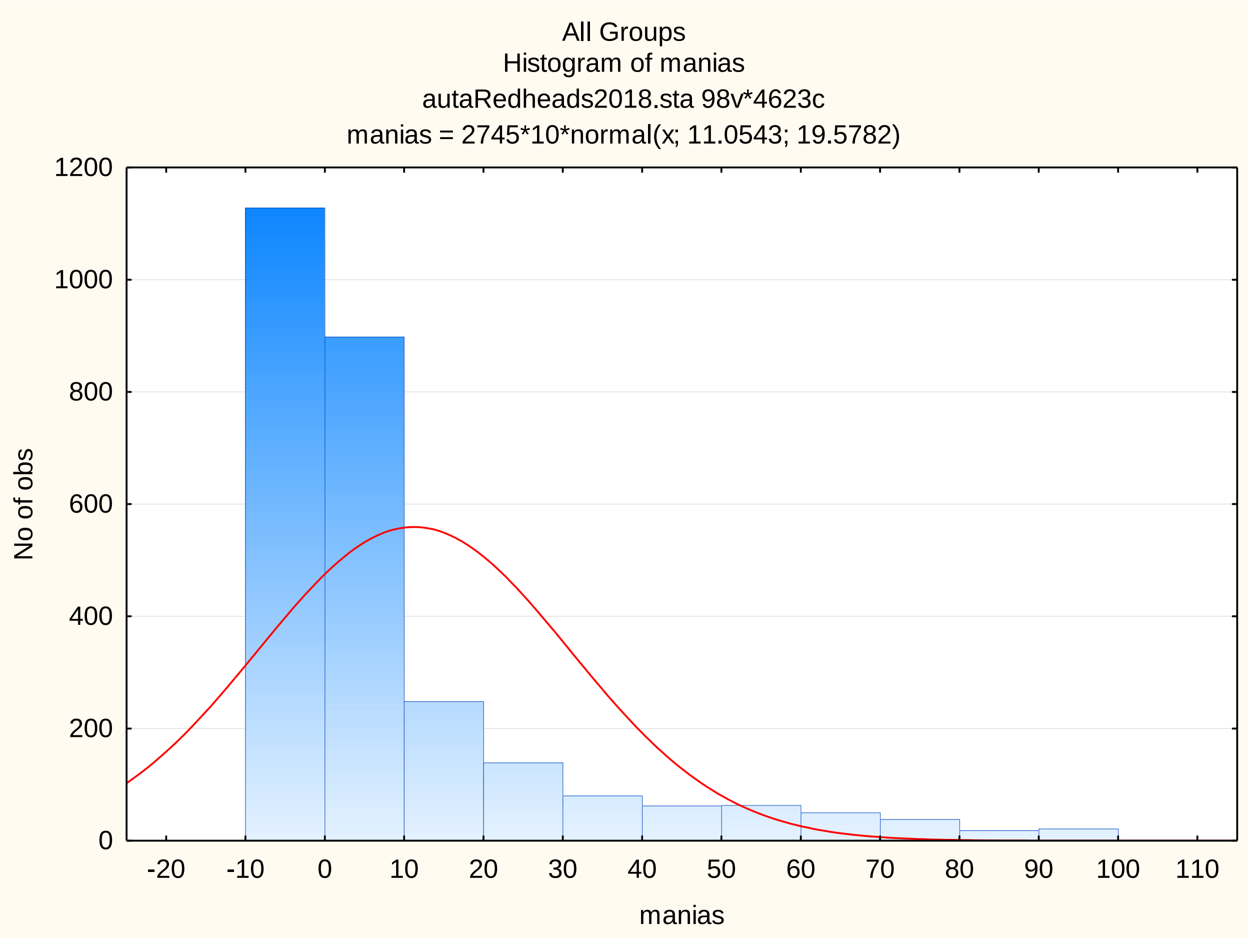
All Groups
Histogram of manias
autaRedheads2018.sta 98v*4623c
manias = 2745*10*normal(x; 11.0543; 19.5782)
No of obs
0
200
400
600
800
1000
1200
-20
-10
0
10
20
30
40
50
60
70
80
90
100
110
manias

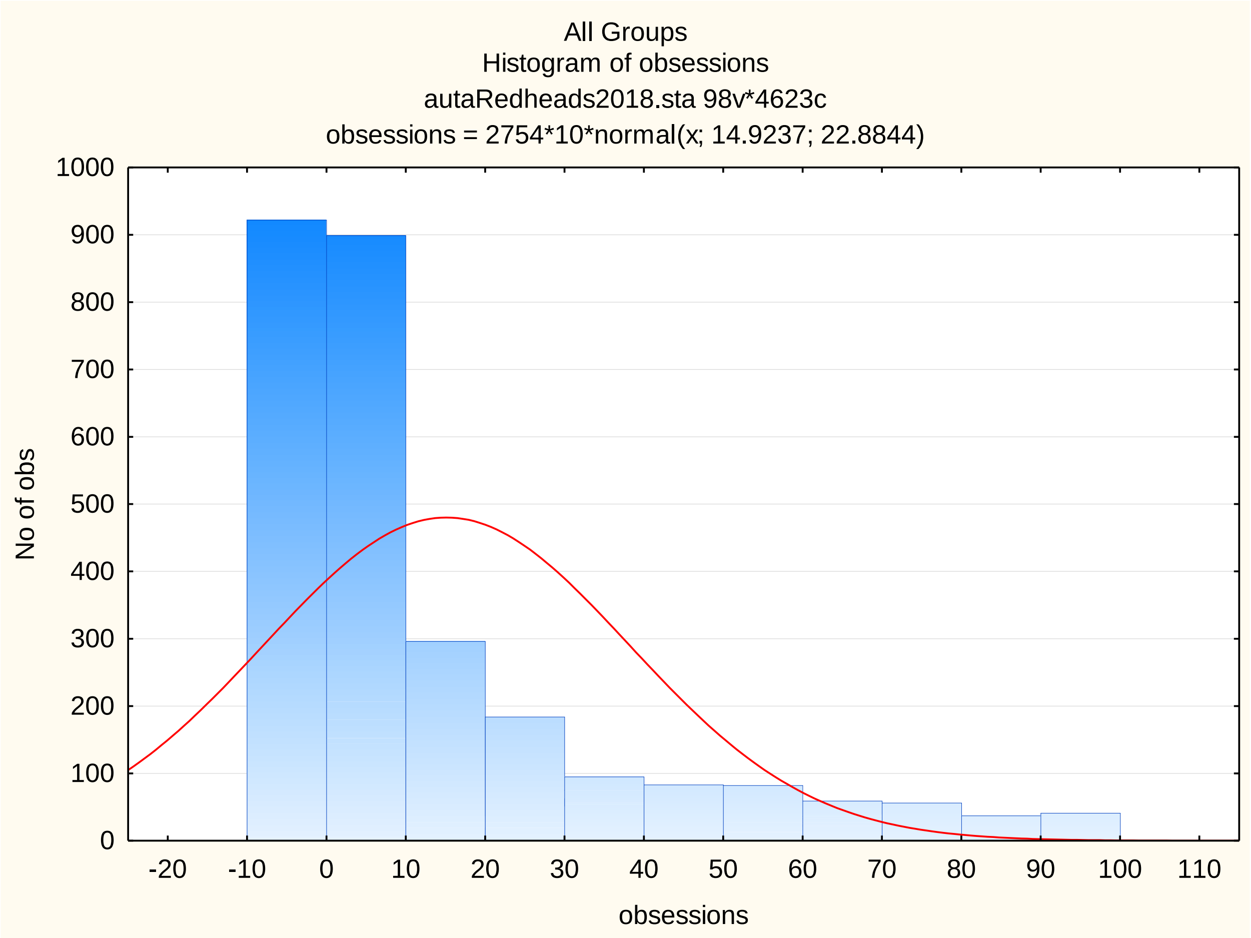
All Groups
Histogram of obsessions
autaRedheads2018.sta 98v*4623c
obsessions = 2754*10*normal(x; 14.9237; 22.8844)
1000
900
800
700
600
500
400
300
200
100
0
No of obs
-20
-10
0
10
20
30
40
50
60
70
80
90
100
110
obsessions

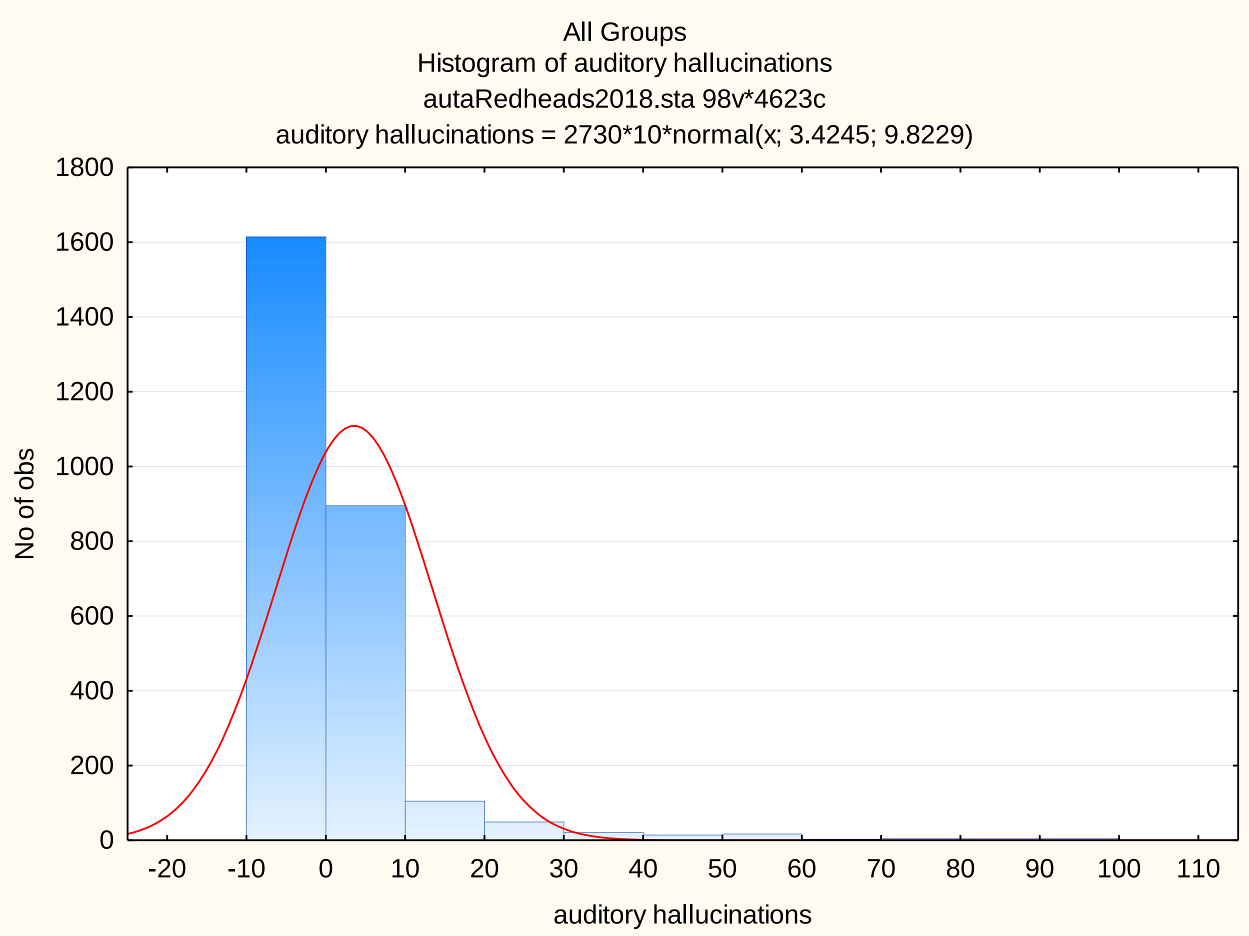
All Groups
Histogram of auditory hallucinations
autaRedheads2018.sta 98v*4623c
auditory hallucinations = 2730*10*normal(x; 3.4245; 9.8229)
No of obs
0
200
400
600
800
1000
1200
1400
1600
1800
-20
-10
0
10
20
30
40
50
60
70
80
90
100
110
auditory hallucinations

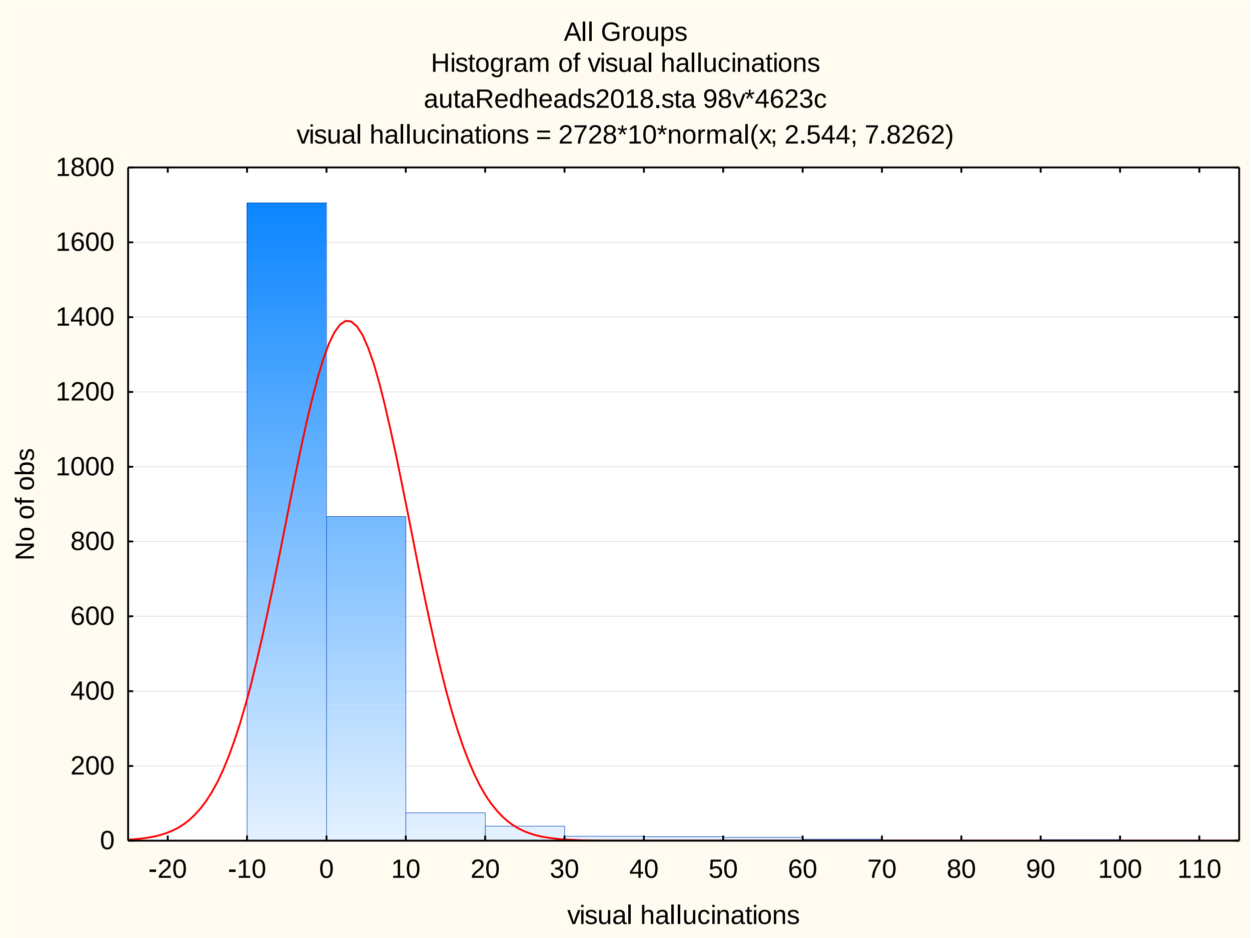
All Groups
Histogram of visual hallucinations
autaRedheads2018.sta 98v*4623c
visual hallucinations = 2728*10*normal(x; 2.544; 7.8262)
No of obs
visual hallucinations
0
200
400
600
800
1000
1200
1400
1600
1800
-20
-10
0
10
20
30
40
50
60
70
80
90
100
110

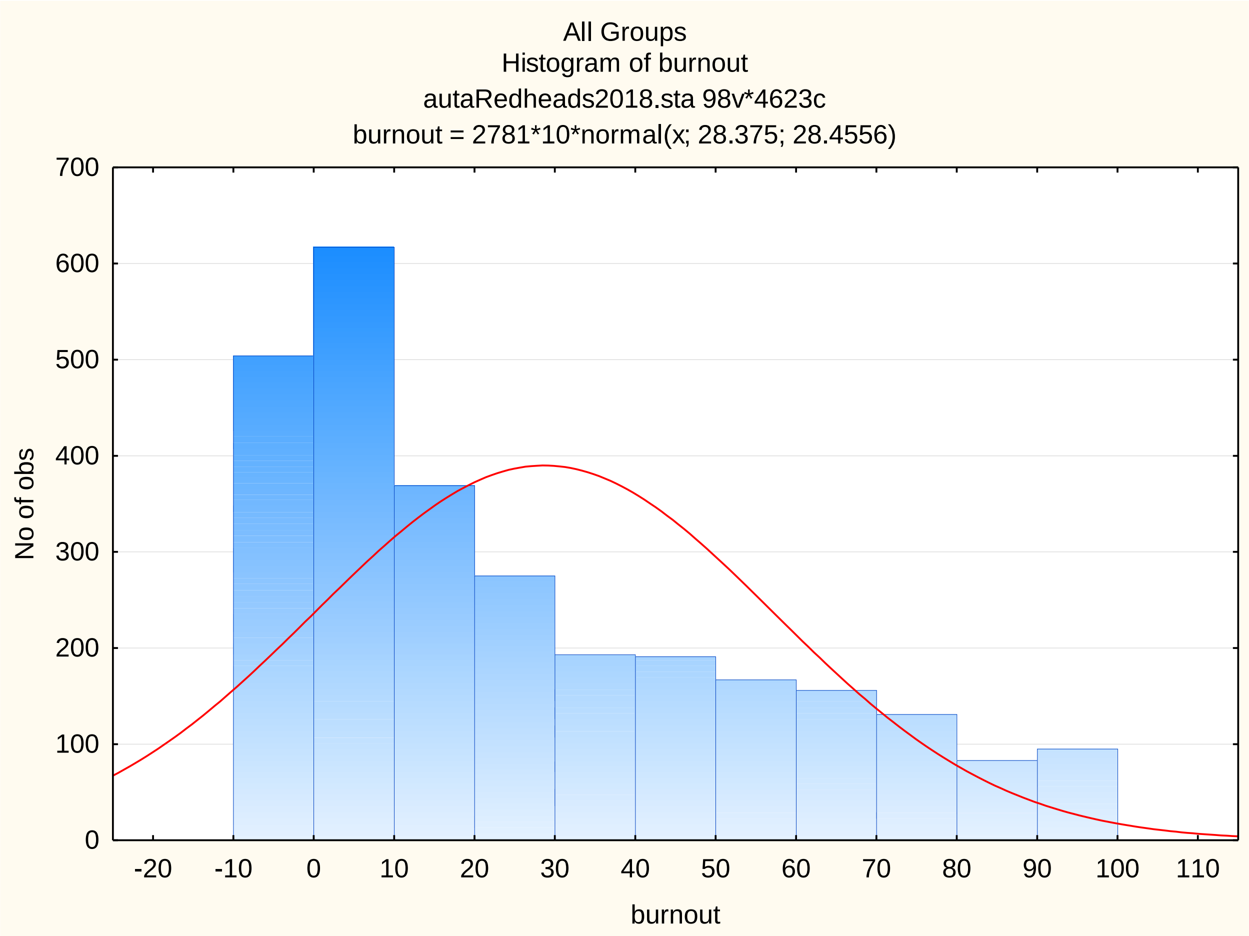
All Groups
Histogram of burnout
autaRedheads2018.sta 98v*4623c
burnout = 2781*10*normal(x; 28.375; 28.4556)
No of obs
0
100
200
300
400
500
600
700
-20
-10
0
10
20
30
40
50
60
70
80
90
100
110
burnout

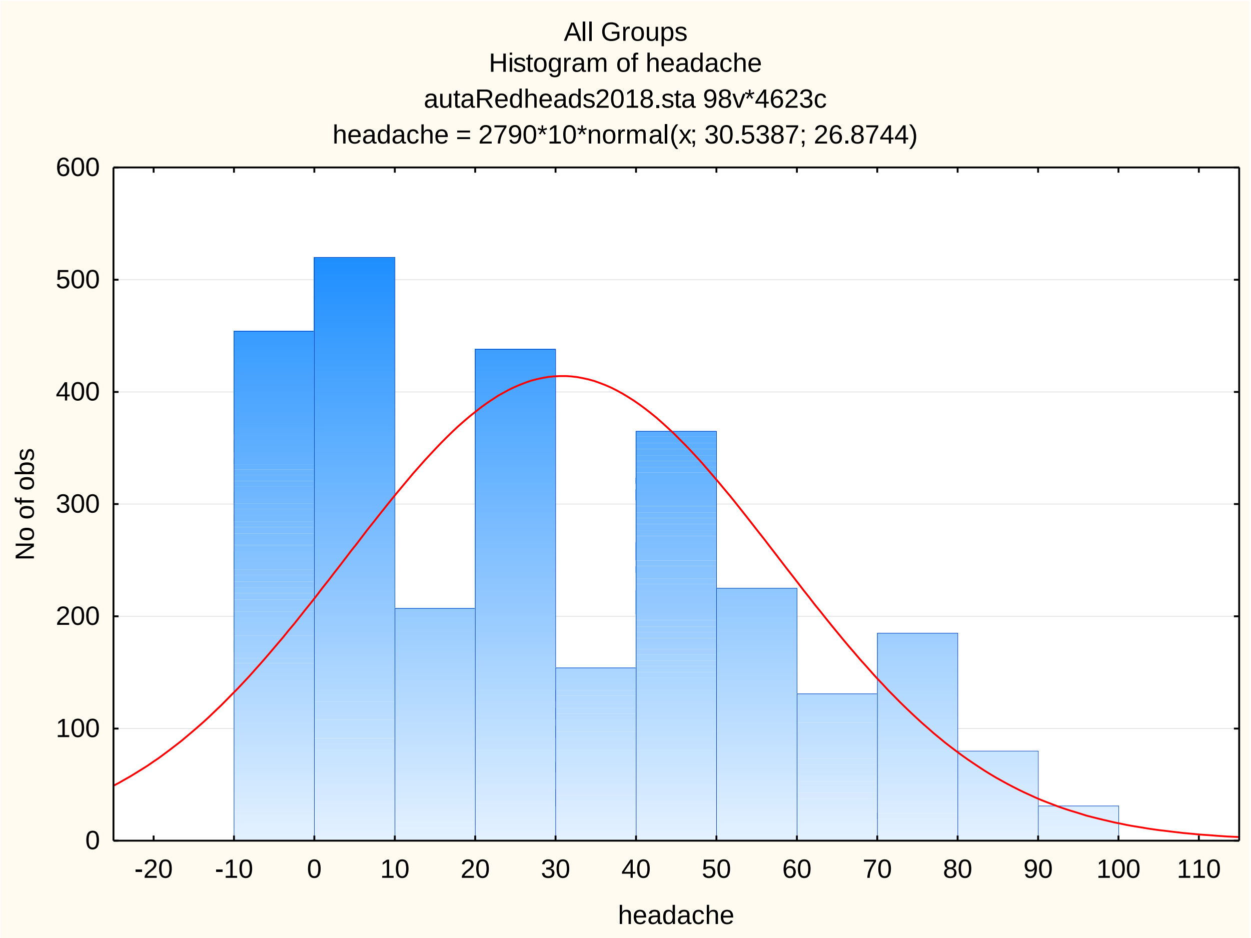
All Groups
Histogram of headache
autaRedheads2018.sta 98v*4623c
headache = 2790*10*normal(x; 30.5387; 26.8744)
No of obs
0
100
200
300
400
500
600
-20
-10
0
10
20
30
40
50
60
70
80
90
100
110
headache

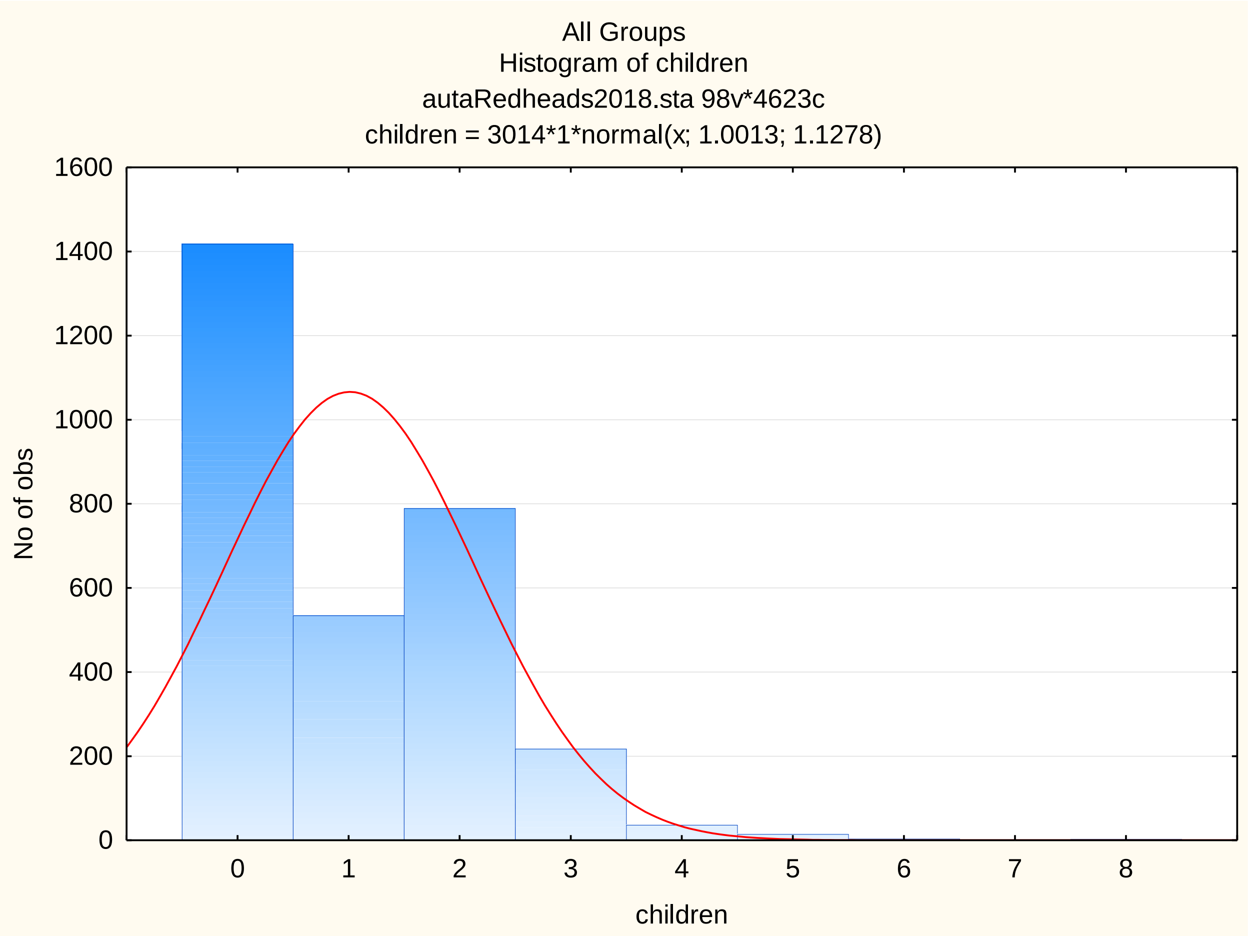

All Groups
Histogram of children
autaRedheads2018.sta 98v*4623c
children = 3014*1*normal(x; 1.0013; 1.1278)
1600
1400
1200
1000
800
600
400
200
0
No of obs
0
1
2
3
4
5
6
7
8
children
